# Determinants of Chinese physicians’ engagement in narrative medicine: a comprehensive SEM-ANN analysis

**DOI:** 10.3389/fmed.2025.1694846

**Published:** 2026-01-15

**Authors:** Jiaqi Li, Zhuomu Hu, Xinyu Gu, Yan Wu, Wei Sun, Yufei Yang, Yexin Hu, Yuxiao Li, Hui Zhang, Hao Zhang

**Affiliations:** 1 Department of Health Policy and Management, School of Public Administration, Hangzhou Normal University, Hangzhou, China; 2 Health insurance office, Zhejiang Province People’s Hospital, Hangzhou, Zhejiang, China; 3 Department of Public Health, School of Public Health, Fudan University, Shanghai, China

**Keywords:** behavioral intention, motivational profiling, narrative medicine, SEM-ANN, theory of planned behavior

## Abstract

**Purpose:**

The implementation of narrative medicine (NM) holds significant implications for multiple stakeholders in healthcare, necessitating investigation into clinicians’ behavioral intention (BI) toward NM practice. This study employs the Theory of Planned Behavior (TPB) to identify predictors of Chinese clinicians’ NM practice intentions, extending the theoretical framework through incorporation of external variables—Perceived Organizational Support (POS) and Perceived Informal Organizational Support (PIOS).

**Patients and methods:**

Data collected from 855 Chinese clinicians validated the theoretical model. The hybrid model of Structural Equation Modeling (SEM) and Artificial Neural Network (ANN) demonstrated superior predictive accuracy, successfully capturing both linear associations and nonlinear interactions among variables. Phase I utilized SEM to identify intention predictors, while Phase II applied ANN to rank predictors’ relative importance. Furthermore, conducting K-means clustering (optimal K = 4 determined by Elbow Method and Silhouette Coefficient) using ATT, SN, PBC, and PIOS as inputs to establish a “Psychological Motivation-Behavioral Intention” coupling profile. Chi-square tests examined distribution differences across organizational contexts.

**Results:**

Perceived behavioral control (PBC) was the strongest BI predictor, followed by PIOS, subjective norms (SN), and attitudes (ATT), with POS being non-significant. Four distinct profiles emerged: “Moderately Engaged, Moderately Intent” (Cluster 1), “Attitudinally Compliant, Structurally suppressed” (Cluster 2), “Cognitively Engaged, Moderately Intent” (Cluster 3), and “Fully Engaged, High-Intent” (Cluster 4). Distribution varied significantly across hospital levels (*χ*^2^ = 22.297, *p* = 0.001) and departments (*χ*^2^ = 26.240, *p* = 0.036), with Cluster 2 predominant in emergency/pediatrics and Cluster 4 dominant in surgery.

**Conclusion:**

ATT, SN, PBC, and PIOS positively influenced physicians’ NM behavioral intention. The motivation-intention coupling profiles reveal heterogeneity in practice potential, offering theoretical-practical insights for targeted interventions.

## 1 Introduction

Health is increasingly understood not as a fixed biological state but as a meaning-laden, socially situated phenomenon shaped by psychological processes, cultural narratives, and interpersonal interactions (1). As the biopsychosocial model gains traction, health is reframed as a contingent, context-dependent construct emerging from the interplay of physiology, lived experience, and cultural discourse (2). Central to this meaning-making process is language: the terms through which illness is described shape how suffering is interpreted, legitimized, and acted upon (3, 4). Within clinical encounters, linguistic framing structures patients’ perceptions of identity, the legitimacy of their symptoms, and the imagined possibilities for recovery. Through diagnostic explanations, treatment narratives, and relational communication, clinicians influence patients’ sense-making in ways that have concrete psychological and behavioral consequences (5, 6). Together, these insights highlight why patient narratives are indispensable for understanding illness experience and why narrative-sensitive practices such as Narrative Medicine (NM) have gained increasing relevance.

However, despite this conceptual shift toward meaning-centered care, contemporary clinical systems often marginalize patients’ subjective narratives. Efficiency-driven workflows reduce opportunities for reflective communication, and non-empathic or ambiguous clinician communication has been linked to heightened patient anxiety, confusion, and reduced agency—ultimately weakening engagement in self-directed health behaviors (7). Cross-cultural studies highlight similar gaps: in China, physicians may underutilize family or informal support networks in chronic disease management (8), while in the UK, most audiologists report never formally assessing patients’ psychosocial states during consultations despite recognizing their importance (9). These patterns suggest a persistent disconnect between the rhetoric of patient-centered care and the structural realities of clinical practice.

NM emerged as a practice-based response to this gap, placing narrative understanding and linguistic attentiveness at the heart of clinical care (10). Originally articulated by Charon, NM emphasizes clinicians’ capacity to “listen to and respond to the stories of the sick” as a core professional competency (11, 12). Narrative practices enable patients to reorganize suffering, reframe identity, and reclaim agency, while clinicians shift from evaluators to co-participants in interpretive dialog (13). Empirical research across the United States, Italy, and China demonstrates NM’s ability to identify unmet psychosocial needs, enhance adaptive capacity, improve quality of life, strengthen therapeutic alliances, and increase patient satisfaction (14–17).

Yet despite this growing evidence base, NM remains peripheral in routine clinical settings, especially within high-pressure systems such as China’s. Although national initiatives like the Action Plan for Enhancing Medical Humanism (2024–2027) formally endorse humanistic care, implementation largely remains limited to fragmented pilot programs (18). Clinicians frequently adopt streamlined communication styles optimized for efficiency and risk control, narrowing the discursive space required for narrative engagement (19–21). Thus, the central challenge is not merely institutional adoption but understanding the motivational and contextual factors shaping clinicians’ willingness to engage in NM under structural constraints.

Given these conditions, clinician engagement with NM should be understood as a behavioral phenomenon shaped by cognitive, motivational, and contextual influences—not solely by skill acquisition. However, existing research predominantly examines patient outcomes (22), educational interventions (23), or program effectiveness (24), with comparatively little attention to the determinants of clinicians’ behavioral intentions. Studies that do explore provider-side engagement rely mainly on descriptive analyses or exploratory interviews (25, 26), lacking a coherent theoretical model to explain how psychological and contextual factors interact.

To meaningfully account for this structural-psychological interplay, a theoretical model is needed that can capture the layered and interacting influences on clinician motivation. The Theory of Planned Behavior (TPB), developed by Ajzen (27), offers a widely validated and parsimonious framework for modeling how behavioral intentions are formed across diverse settings. According to TPB, intention is shaped by three interrelated cognitive components: (a) one’s evaluation of the behavior (attitude), (b) perceived social expectations (subjective norm), and (c) perceived control over performing the behavior (perceived behavioral control). The TPB framework has been extensively applied and empirically supported in domains such as health promotion, clinical decision-making, and professional training—demonstrating its utility in modeling complex, intention-related behaviors (28–30).

Recent scholarship increasingly advocates extending TPB to address its limitations in explaining professional behaviors that occur within complex organizational environments. TPB primarily focuses on intra-individual psychological determinants, while the external contextual forces that shape these beliefs remain largely implicit within the model (31). In practice, however, behavioral intention does not emerge in a vacuum; it is deeply embedded in institutional arrangements, resource constraints, and social networks. For NM—a clinically innovative practice that depends heavily on contextual interaction, reflective engagement, and emotional investment—individual cognitions alone are insufficient to fully explain clinicians’ adoption decisions (32). Accordingly, incorporating key structural and contextual variables into TPB has become a common extension in organizational and health behavior research. Such an expansion does not negate the core logic of TPB but enriches it by introducing “external antecedents” that enhance the model’s contextual fidelity and predictive depth. The present study focuses on two complementary constructs—Perceived Organizational Support (POS) and Perceived Informal Organizational Support (PIOS)—chosen based on a systematic analysis of the dual characteristics of the Chinese healthcare system. First, China’s public medical institutions are characterized by strong bureaucratic structures and policy-driven governance. Formal arrangements—such as resource allocation, performance appraisal, and administrative directives—constitute powerful and direct contextual forces shaping clinicians’ work. Second, Chinese sociocultural norms are deeply rooted in relationalism and collectivism, which are amplified in clinical environments that emphasize teamwork and interdependence. The core practices of NM—empathic communication, reflective listening, and narrative meaning-making—are not easily transmitted through formal documentation but are instead learned through interpersonal relationships, modeling, and experiential exchange.

In this context, integrating POS and PIOS provides a theoretically meaningful dual-pathway extension of TPB. POS captures the *top-down institutional push*, while PIOS represents the *horizontal, network-based pull* of social learning and peer support. Together, these constructs allow the extended TPB model to more accurately and holistically reflect the motivational landscape faced by Chinese clinicians when considering the adoption of NM—a complex, relational, and context-dependent clinical practice.

Methodologically, this study employs a rigorous hybrid analytical framework by integrating Structural Equation Modeling (SEM) and Artificial Neural Networks (ANN). SEM is utilized for its theory-driven capacity to dissect direct, indirect, and mediating effects among latent constructs, enabling hypothesis testing grounded in established behavioral theories (33). However, given that physicians’ engagement intentions are influenced by multifaceted and interacting psychological and organizational determinants, a purely linear modeling approach may fail to uncover nonlinear or higher-order effects—particularly those reflecting the moderating role of contextual supports on cognitive drivers. To address this limitation, ANN is introduced as a complementary, data-driven technique, known for its proficiency in modeling nonlinear dynamics, detecting intricate multidimensional interactions, and approximating complex functional relationships without requiring strict distributional assumptions (34). The integration of ANN is not merely supplementary but strategically essential, allowing for a more comprehensive and nuanced understanding of the underlying mechanisms in a psychosocial context (35). This dual-method strategy enhances both the explanatory power and predictive robustness of the findings, thus ensuring methodological sophistication and empirical depth.

## 2 Research hypothesis

### 2.1 Attitude

The TPB defines attitude (ATT) as an individual’s overall evaluative judgment about a specific behavior. In this study, physicians’ attitude toward NM refers to their affective and cognitive evaluation of NM as a meaningful and worthwhile clinical activity.

A growing body of TPB-based research has consistently demonstrated that attitude serves as a foundational determinant of behavioral intention, functioning as a primary cognitive-affective anchor that shapes individuals’ motivational readiness to act, particularly in professional healthcare contexts. For example, Rashidian and Russell (36) found that UK general practitioners’ attitudes significantly predicted their intention to adopt prescription guidelines. Similarly, Wiese et al. (37) showed that Irish physicians’ attitudes toward maintaining professional competence were strongly associated with their commitment to continued professional development. Rich et al. (38) reported that physicians’ attitudes toward reflective practice and confidentiality guidelines were the strongest predictors of intention to engage in those behaviors. In the Chinese context, Wang et al. (39) found that primary care physicians’ attitudes significantly influenced their willingness to engage in shared decision-making with patients suffering from acute respiratory infections.

Taken together, these findings suggest that clinicians’ attitudinal stance toward a particular practice plays a critical role in their behavioral intention. Although few studies have directly examined NM, prior evidence strongly indicates that physicians’ attitudes are likely to shape their willingness to adopt narrative approaches in clinical settings.

*H1*: Physicians’ ATT toward NM will positively predict their intention to engage in NM practice.

### 2.2 Subjective norm

Within the framework of the TPB, Subjective norm (SN) refers to the perceived social pressure individuals experience from salient others when considering whether to perform a particular behavior. In the context of NM, such normative expectations may arise from patients, institutional stakeholders, governmental policies, and educational authorities.

A growing body of research has affirmed the predictive value of SN in shaping clinicians’ behavioral intentions across diverse healthcare contexts. Patients, as core recipients of clinical accountability, have been established as primary SN sources in prior research: A multicenter survey of UK pediatricians by O’Connell et al. (40), found that physicians perceiving stronger expectations from patients and families showed greater propensity to refer children to psychological services. In China, a cross-sectional study of physicians in county-level medical alliances by Zhao et al. (41) demonstrated SN as the strongest predictor of referral intentions. At the governmental level, research conducted in China identified SN derived from the “Consistency Evaluation Policy” and national insurance payment regulations as significant predictors of prescribing intentions for high-quality generic drugs (42). Institutional influence was evidenced through a cross-sectional survey in Chinese county hospitals, proving that policy-reinforced normative requirements (e.g., Essential Medicines List) significantly enhanced physicians’ intention to prioritize generic drugs (43). Educator norms also demonstrated impact, as observed in a study on medical students’ behavioral intentions toward learning AI in clinical practice, wherein faculty encouragement increased willingness to engage in AI-related learning behaviors (44).

While few studies have directly examined SN in relation to NM practice, these findings collectively suggest that perceived social expectations constitute a salient motivational factor for clinicians when adopting behaviors framed as ethically or institutionally normative.

*H2*: SN will positively predict physicians’ intention to engage in NM practice.

### 2.3 Perceived behavioral control

Perceived behavioral control (PBC), a core TPB construct, reflects an individual’s self-assessment of their capability to perform a specific behavior and anticipated barriers/facilitators. In this study, physicians’ PBC represents their self-perceived competence in implementing NM and achieving desired clinical outcomes. Empirical evidence consistently highlights PBC’s critical role in shaping behavioral intentions, particularly among healthcare professionals. A cross-sectional study conducted among Chinese physicians revealed that PBC positively predicts primary care physicians’ intention to provide cervical cancer screening services for rural women in China (45). Likewise, a systematic review exploring physicians’ willingness to share knowledge also highlights PBC as a strong predictor of behavioral intentions (46). Another study conducted in Italy further identified PBC as a significant predictor of physicians’ intention to transfer MOOC-acquired knowledge and skills to clinical practice, demonstrating stronger predictive power than other TPB constructs (47). In addition, a Canadian cross-sectional study of specialists completing web-based Continuing Professional Development courses demonstrated a robust association between physicians’ self-efficacy and their willingness to adopt innovative clinical behaviors (48). Given NM’s emerging status in many clinical settings, PBC likely plays a pivotal role in its adoption process. When physicians perceive competence in integrating NM into clinical routines and overcoming implementation barriers, their NM adoption intentions may strengthen. Thus, we hypothesize:

*H3*: PBC positively influences physicians’ intention to practice NM.

### 2.4 Perceived organizational support

POS is grounded in organizational support theory and refers to employees’ perceptions of the extent to which their organization values their contributions and cares about their well-being (49). In practice, POS is manifested through various forms of formal, tangible support—such as protected time, funding, performance evaluation systems, incentive structures, and leadership behaviors. These signals collectively convey multidimensional institutional support originating from the formal organizational hierarchy.

In health services research, POS has been widely employed to explain how institutional environments shape professionals’ adoption of, or sustained engagement in, practices aligned with organizational values. In the specific context of NM, POS captures clinicians’ perception of whether their institution recognizes and supports NM-related activities as a legitimate and valued component of professional care. POS often functions as a normative institutional signal—communicated through performance metrics, incentive mechanisms, workload adjustments, or leadership endorsement—indicating that NM practices are expected, encouraged, and organizationally sanctioned.

Existing research provides empirical support for this association. For instance, an online survey of nurses and physicians in the United States found that POS motivated healthcare professionals to correct health misinformation on social media (50). Similarly, a study of nurses in 19 tertiary hospitals in the Philippines showed that POS was a significant predictor of smartphone use in clinical tasks (51). Within the NM field, formal organizational support often takes the form of normative reinforcement measures, which are considered foundational conditions for clinicians’ participation in NM practice (52).

However, POS may be impeded by several contextual constraints. Heavy clinical workload, limited time availability, and insufficient awareness or acceptance of NM among institutional leaders can hinder implementation efforts and weaken clinicians’ motivation to engage in NM. Many healthcare professionals report dissatisfaction with current levels of organizational support, noting discrepancies between institutional expectations and the actual resources or conditions available to support such practices. These concerns have led to growing calls for institutional improvement and more meaningful organizational support (53).

On the basis of this theoretical and empirical foundation, we propose the following hypothesis:

*H4*: POS has a positive effect on clinicians’ intention to implement NM.

### 2.5 Perceived informal organizational support

PIOS refers to an individual’s perception of non-institutional, peer-based support that emerges from social networks, shared norms, and informal learning environments. Unlike formal organizational structures, informal support arises spontaneously through interpersonal interactions and organically formed professional communities (54). As a contextual extension of TPB, PIOS illuminates how broader social structures within healthcare organizations shape intention formation beyond formal administrative channels.

Social Learning Theory (SLT) and Communities of Practice (CoP) provide the principal theoretical foundations for understanding PIOS. SLT posits that individuals develop behavioral intentions by observing and interacting with trusted peers and mentors (55). A systematic review on mentorship in healthcare indicates that informal peer support and role-modeling are key predictors of adopting innovative practices (56). Consistently, Bautista et al. (51) found that healthcare professionals who received informal support from colleagues or academic networks reported stronger intentions to engage in new behaviors.

CoP theory further demonstrates how collaborative professional groups shape clinical behaviors by fostering multidisciplinary interaction, enhancing cross-boundary learning, and strengthening shared professional identity (57). Because NM relies heavily on experiential knowledge, reflective dialog, and narrative sense-making, CoP mechanisms—such as storytelling, case reflection, and shared meaning construction—are particularly valuable (58).

In the context of NM, PIOS may manifest through mentor guidance, peer modeling in narrative encounters, cross-institution NM workshops, professional discussion groups, or informal NM-related conversations during clinical case exchanges or online learning communities.

Importantly, these informal mechanisms exert particularly strong influence in Chinese clinical settings. Cultural psychology and organizational behavior research show that in collectivist and relational cultures, peer expectations, interpersonal norms, and group endorsement often shape professional behaviors more directly than formal administrative directives (59, 60). Additionally, the clinical microsystem perspective highlights that daily behavioral routines in hospital departments are shaped more by local team culture than by higher-level institutional policies (61). Within such contexts, informal networks provide clinicians with credible, context-sensitive, and immediately actionable cues—making PIOS a potentially more salient motivational force than formal support.

Based on this theoretical foundation, we propose the following hypothesis:

*H5*: PIOS has a positive effect on clinicians’ intention to implement NM.

### 2.6 Practice behavior

Behavioral intention (BI) serves as one of the strongest predictors of actual behavior, particularly when individuals possess sufficient control capabilities and environmental support enables behavioral execution (27). In the context of NM, practice intention reflects healthcare professionals’ preparedness and commitment to adopting NM. A systematic review investigating healthcare professionals’ adoption of novel practices found intention-behavior correspondence sufficiently strong to warrant intention as a valid proxy measure (62). Quantitative meta-analyses estimate that 28% of behavioral variance (*R*^2^) is attributable to intention (63). In addition, another systematic review identifies BI as the strongest predictor of medical humanities practice adoption, such as shared decision-making (64). Therefore, we hypothesize that clinicians with stronger intentions to engage in patient-centered practices—such as NM—are more likely to translate those intentions into concrete actions. Thus, this study postulates:

*H6*: Doctors’ intention to practice NM positively influences their practice behavior.

**Figure 1:**
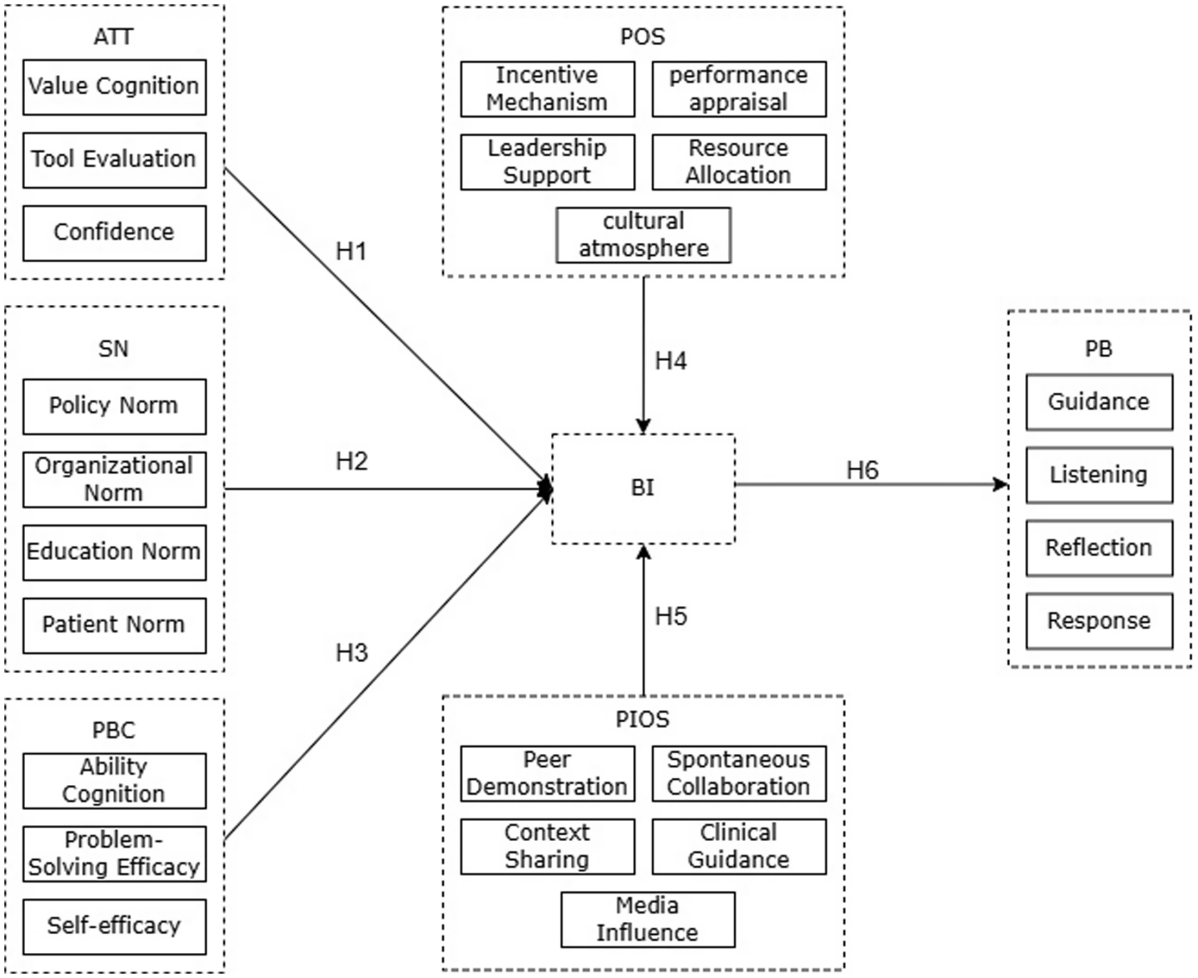
The proposed research model diagram.

Based on the above, the theoretical model was proposed in this study (see Figure 1).

## 3 Materials and methods

### 3.1 Study design

This study employed a cross-sectional, multi-site survey design to empirically test an extended TPB model predicting clinicians’ intention to adopt NM. Grounded in TPB, the study aimed to capture not only individual-level cognitive antecedents—attitudes, subjective norms, and perceived behavioral control—but also contextual variables, namely POS and PIOS, theorized to shape intention formation in institutionally constrained environments. Data were collected from physicians practicing in provincial, municipal, and county-level hospitals across Zhejiang Province, encompassing a broad range of institutional structures and socioeconomic contexts. A stratified sampling strategy ensured representation across different organizational tiers. A total of 1,000 structured questionnaires were administered via both online and offline platforms. The survey instrument assessed clinicians’ demographic and professional characteristics, their beliefs and attitudes toward NM, perceived social expectations, perceived behavioral control, and perceived institutional and interpersonal support. By embedding TPB constructs within real-world organizational strata, the design enabled examination of how multilevel forces interact to shape behavioral intention. In doing so, the study operationalized intention as a function of both internal motivation and external structural conditions. Rigorous quality control procedures—including logic checks, attention filters, and exclusion of incomplete responses—were implemented to ensure the validity and reliability of the data.

### 3.2 Sampling strategy

To achieve contextual heterogeneity and theoretical alignment with the extended TPB, we employed a dual-layered stratified sampling strategy based on both horizontal (regional socioeconomic strata) and vertical (institutional hierarchy) dimensions (see Table 1). Zhejiang Province was selected as the study site due to its pronounced variation in economic development, healthcare accessibility, and geographic characteristics. This heterogeneity offers an ideal empirical setting to examine how institutional and environmental conditions shape clinicians’ behavioral intentions toward NM. Using cluster analysis on multiple socio-economic indicators—including GDP per capita, fiscal capacity, industrial composition, healthcare infrastructure, and geographic accessibility—we stratified prefectures into three economic tiers:

(1) Affluent regions included Hangzhou (the provincial capital) and Ningbo (a key coastal port), both representing high-resource, institutionally mature urban hubs.
(2) Moderately affluent areas comprised Taizhou, Shaoxing, Wenzhou, Huzhou, and Jiaxing, which exhibit relatively stable economic profiles and expanding medical systems.
(3) Less affluent regions consisted of Lishui and Quzhou, characterized by mountainous terrain, limited connectivity, and constrained healthcare capacity.

**Table 1:** Distribution of sampled clinicians by hospital level and socioeconomic region.

Hospital level	Affluent region	Moderately affluent region	Less affluent region	Total
County-level Hospitals	97	195	56	348 (41.0%)
Municipal-level Hospitals	115	230	65	410 (48.0%)
Provincial-level Hospitals	97	–	–	97 (11.0%)
Total	309 (36.1%)	425 (49.7%)	121 (14.2%)	855 (100%)

In parentheses is the ratio of the number of components to the total number.

Clinician participants were proportionally recruited across these strata: 28% from affluent regions, 56% from moderately affluent regions, and 16% from less affluent regions. This allocation reflects the demographic and institutional distribution of the physician workforce across the province. Within each regional tier, we further sampled hospitals at three institutional levels—provincial, municipal, and county-level—to capture organizational diversity. This vertical stratification mirrors Zhejiang’s physician composition: approximately 41% of physicians work in county-level hospitals, 48% in municipal institutions, and 11% in provincial centers. These proportions were used to guide sample quotas, enhancing ecological validity. By integrating regional economic context with organizational hierarchy, this dual-stratified sampling strategy enables a comprehensive analysis of how macro-structural forces and meso-level institutional environments jointly interact with individual-level cognitive and motivational variables. It thus provides the necessary empirical foundation for testing the extended TPB framework and understanding intention formation toward NM in real-world clinical ecologies.

### 3.3 Procedure and control

Data were collected using a mixed-mode approach, combining online questionnaires with on-site paper administration across participating hospitals. All participants provided informed consent prior to data submission. To enable data validation and longitudinal linkage while protecting confidentiality, phone numbers and national ID numbers were securely collected and stored in encrypted form, accessible only to authorized personnel. Individual-level data were anonymized prior to analysis.

To minimize response duplication, online surveys were limited to one submission per IP address and device. Paper-based responses were monitored by trained research assistants to ensure participant engagement and compliance with the protocol. Following collection, all questionnaires underwent a rigorous quality control process. Dual data entry was employed to reduce transcription errors, and logical cross-checks were performed to identify invalid responses.

The following exclusion criteria were applied: (1) incomplete surveys (missing >20% of items), (2) excessive response uniformity across Likert scales (e.g., straightlining >90%), (3) completion times under 5 min, and (4) internally contradictory responses. Any flagged entries were subject to re-verification through participant re-contact or manual review. Only valid and verified responses were retained for final analysis.

### 3.4 Participant demographics

In December 2023, a total of 1,000 questionnaires were distributed to practicing physicians across Zhejiang Province. After data validation, 855 valid responses were retained (response rate = 85.5%). The final sample encompassed a wide range of sociodemographic and professional profiles, ensuring analytic representativeness (see Table 2). Gender was nearly balanced (53.33% male; 46.67% female), and the majority of respondents were between 30 and 49 years of age, aligning with the age structure of China’s clinical workforce. Educational attainment was high, with 95.56% holding a bachelor’s degree or above, including 32.28% with a master’s or higher degree. Professional seniority followed a graded distribution: 27.49% held a primary title, 29.82% a middle title, 24.44% an associate senior title, and 18.25% a senior title. Clinical experience was similarly varied, with 34.15% having 5–14 years of experience and 42.45% with 15 or more years, reflecting a professionally mature cohort. Respondents came from a range of clinical departments. Internal medicine and surgery accounted for 56.14% collectively, followed by emergency, critical care, pediatrics, obstetrics-gynecology, and other specialties. This structure mirrors the departmental distribution typical of Chinese hospitals. Institutionally, 76.73% were employed at general hospitals and 23.27% at specialized hospitals. Monthly income varied, with 47.60% earning between 5,000–9,999 CNY, and 14.15% earning over 15,000 CNY. This income gradient roughly corresponds to hospital tier, seniority, and regional economic conditions. Thus, the sample reflects substantial heterogeneity in personal, professional, and institutional domains, providing a robust empirical foundation for modeling clinicians’ intention to engage in NM.

**Table 2:** Sociodemographic characteristics of participants (*N* = 855).

Characteristic	Category	*n*	%
Gender	Male	456	53.33%
	Female	399	46.67%
Age(years)	<30	176	20.58%
	30~	268	31.35%
	40~	294	34.39%
	50~	117	13.68%
Marital status	Unmarried	189	22.11%
	Married	652	76.26%
	Divorced / other	14	1.64%
Educational Level	Junior college or below	38	4.44%
	Bachelor’s degree	541	63.27%
	Master’s degree or above	276	32.28%
Professional title	Primary title	235	27.49%
	Intermediate	255	29.82%
	Associate senior title	209	24.44%
	Senior title	156	18.25%
Monthly income (CNY)	<5,000	104	12.16%
	5,000~	407	47.60%
	10,000~	223	26.08%
	15,000~	121	14.15%
Clinical department	Emergency and critical care medicine	65	7.61%
	Internal medicine	300	35.09%
	Surgery	180	21.05%
	Pediatrics	32	3.74%
	Obstetrics and gynecology	36	4.21%
	Others	242	28.30%
Hospital Type	General hospital	656	76.73%
	Specialized hospital	199	23.27%
Years of experience	<5	200	23.39%
	5~	292	34.15%
	15~	229	26.78%
	25~	134	15.67%

### 3.5 Ethical approval

Prior to the commencement of data collection, participants were apprised of the study’s objectives and methodologies. It was clearly communicated that participation was entirely voluntary, and that their data would be anonymized to safeguard privacy, used solely for research endeavors. To acknowledge participants’ time and contribution, a modest, ethics-approved compensation was provided. Participation was fully voluntary; participants could withdraw at any time without penalty, and the compensation was not contingent on completing the survey/interview. The research received approval from the Ethics Committee of Hangzhou Normal University (approval number: 20230001). Adhering to the ethical standards delineated in the 1964 Declaration of Helsinki, including subsequent revisions, the study ensured that each participant provided informed consent.

### 3.6 Measures

The design and refinement of the survey questions can be delineated into a three-stage process, comprising: (1) literature review and scale translation, (2) qualitative interviews and thematic analysis, and (3) a pilot study to finalize the instrument.

Stage 1: literature review and translation.

The initial phase involved a comprehensive review of existing instruments based on the TPB, as well as previously validated measurement tools related to NM practice. Specific item references are detailed in Supplementary Table 1. Core TPB constructs—including Attitude, Subjective Norm, and Perceived Behavioral Control—were drawn from Ajzen (27)‘s original work and subsequently translated into Chinese to ensure linguistic and cultural appropriateness for Chinese physicians. Furthermore, relevant research on NM was also reviewed to ensure that the unique features of NM—such as the importance of empathetic communication, patient storytelling, and the co-construction of meaning in healthcare—were integrated into the development of the measurement scales. In particular, studies by Charon (65) have highlighted the value of narrative competence in clinical practice and its impact on patient outcomes, which influenced the adaptation of the scales for this context. To enhance translation fidelity, a two-step process was followed: initial translation using professional software, followed by manual revision and cross-checking by native Mandarin speakers fluent in English. This process ensured that both the TPB constructs and the NM-related dimensions were accurately represented in the Chinese context.

Stage 2: semi-structured interviews and thematic analysis.

Subsequently, although a literature review was conducted to gather a wide range of measurement frameworks, evaluation tools, and assessment indicators, qualitative methods were also necessary to incorporate more localized elements such as unique historical, cultural, social, economic, and healthcare system characteristics specific to China. In this study, semi-structured open-ended interviews were utilized to elicit detailed descriptions of experiences and insights from both doctors and service recipients. Semi-structured interviews were conducted with 152 healthcare professionals and administrators, and thematic analysis based on grounded theory was applied to the data to identify measurement indicators that are both universally applicable to the Theory of Planned Behavior and specifically relevant to NM. The selection of interviewees was achieved through a combination of stratified sampling and purposeful sampling. Stratified sampling was based on economic development levels, targeting seven provinces across eastern, central, and western China, and purposeful sampling ensured diversity among participants by including individuals of different genders, ages, professions, and educational backgrounds. The interviews, conducted between July and September 2023, were led by eight researchers specialized in health management, utilizing both in-person and telephone interviews. The interview guide was continuously refined based on a literature review until theoretical saturation was reached. After obtaining informed consent, each interview, lasting over 30 min, was audio-recorded and transcribed. Thematic analysis was performed, with NVivo 11 software aiding in initial coding. Two coders discussed and determined the themes, while a third researcher resolved coding disputes. Themes were subsequently mapped onto the literature review results, informing the development of the PB scale, POS scale, and PIOS scale. Based on the qualitative insights, the original ATT, SN, and PBC scales were further revised and localized for the Chinese context. The corresponding First-level Descriptive Themes, Second-level Analytic Themes, Third-level Analytic Themes, frequencies, and original data examples are presented in Supplementary Table 2.

Stage 3: pilot testing and finalization.

Before formal data collection, a pilot study was conducted to test the clarity, reliability, validity, and cultural appropriateness of the revised instrument. A total of 198 physicians completed the pilot survey. Based on item-level feedback and response patterns, we convened focused group discussions to identify items that were ambiguous, difficult to understand, or contextually inappropriate, and revised the wording accordingly to finalize the questionnaire. All items were measured on a 7-point Likert scale (1 = “strongly disagree” to 7 = “strongly agree”), which is commonly used in psychosocial research to improve response sensitivity and reduce ceiling/floor effects. Supplementary Table 1 presents all measurement items, listing indicators for each construct along with their original source or rationale for adaptation.

#### 3.6.1 Attitude

The ATT scale was developed as a standardized tool to systematically assess clinicians’ cognitive attitudes, value judgments, and developmental expectations toward NM. After a thorough literature review, key constructs from Ajzen’s (27) theory of attitude were integrated with attitude subdimensions identified in Chinese-context studies—specifically, KAP (knowledge-attitude-practice) research on narrative nursing by Jia et al.’s (66) KAP research on NM by Guo et al. (26); and research on narrative literacy by Yang et al.’s (67). These sources informed initial indicators such as recognition of NM’s value and perspectives on its future development. Semi-structured interviews were then conducted to refine six localized thematic lexicons: people-oriented (emphasizing patient-centered care), humanistic concern in medicine (ethical practice), practicality (implementation challenges), acceptance (receptiveness to the concept), potential for development* (capacity-building), and future perspective (disciplinary foresight). The finalized instrument consists of three theoretically grounded dimensions. (1) Core Value Recognition: Incorporating the themes of “people-oriented” and “humanistic concern in medicine” to evaluate the ethical necessity of NM. (2) Practical Feasibility Appraisal: Integrating “practicality” and “acceptance” to measure resistance in clinical implementation. (3) Developmental Trajectory Projection: Combining “potential for development” and “future perspective” to quantify expected benefits.

#### 3.6.2 Subjective norm

The SN scale is designed to assess clinicians’ perceptions of social influences from key stakeholders regarding the adoption of NM practices. The development of the scale began with a systematic review of literature, integrating measurement components from the original TPB framework and SN-related factors identified in the study by Cheng and Wang (68) on influences affecting narrative nursing practice among clinical nurses. Grounded theory interviews were then conducted to extract eight core macro-level themes: “policy advocacy” and “institutional support” representing government orientation; “managerial promotion” and “practice encouragement” reflecting organizational behavior; “curriculum integration” and “teaching innovation” relating to the educational system; and “communication needs” and “humanistic expectations” derived from patient demands. Based on these themes, a formal scale was constructed with four validated dimensions: (1) Policy Norms, combining “policy advocacy” and “institutional support” to quantify the implementation pressure from health authorities; (2) Organizational Norms, integrating “managerial promotion” and “practice encouragement” to assess the expectations conveyed by hospital management; (3) Educational Norms, merging “curriculum integration” and “teaching innovation” to evaluate the competency requirements imposed by medical education systems; and (4) Patient Norms, linking “communication needs” and “humanistic expectations” to reflect patients’ emotional expectations for narrative communication.

#### 3.6.3 Perceived behavioral control

The PBC scale is designed to assess clinicians’ self-perceived capability and controllability in implementing NM practices. The development process began with a theoretical grounding in Ajzen’s concept of PBC and theory of self-efficacy by Schwarzer et al. (69), while also incorporating PBC-related factors identified by Cheng and Wang (68) in their study on factors influencing narrative nursing practice among clinical nurses. Based on qualitative interview data, six core thematic terms were distilled: “practical competence” (assessing technical proficiency), “technical mastery” (evaluating command of narrative tools), “adaptability” (coping with unexpected clinical scenarios), “analytical thinking” (interpreting complex narratives), “level of confidence” (reflecting self-assurance), and “motivational persistence” (sustaining long-term engagement). These themes were further structured into three validated second-order dimensions: (1) Cognitive Competence, which combines “practical competence” and “technical mastery” to assess clinicians’ foundational NM skills; (2) Problem-Solving Efficacy, integrating “adaptability” and “analytical thinking” to evaluate the ability to manage narrative conflicts in clinical settings; and (3) Self-Regulatory Efficacy, merging “level of confidence” and “motivational persistence” to quantify resilience in the face of practice-related setbacks. Each dimension comprises multiple validated items that are systematically aligned with the underlying theoretical constructs.

#### 3.6.4 Perceived organizational support

The development of the POS scale aims to capture the multidimensional characteristics of organizational support for NM practice within the Chinese healthcare system. Grounded in Organizational Support Theory (OST) proposed by Eisenberger et al. (70) and informed by relevant empirical studies in Chinese clinical contexts—such as Li et al.’s (71) research on the relationship between POS, well-being, and narrative competence among clinical nurses, and Liu et al.’s (72) study on POS and caring behavior among outpatient nurses—the scale integrates theoretical and contextual perspectives. Based on qualitative interviews, 10 localized thematic indicators were identified: reward strategies (performance bonuses for NM practice), recognition practices (internal awards for narrative cases), assessment orientation (promotion criteria weighting), metric design (specific evaluation standards), advocacy behavior (public endorsement by leadership), modeling by example (dissemination of exemplary clinician stories), infrastructure support (facilities and space provision), financial support (dedicated budget allocations), humanistic atmosphere (narrative culture within departments), and narrative orientation (strategic inclusion of NM in hospital planning). These were further organized into 5 sec-order dimensions: (1) Incentive Mechanisms, combining reward strategies and recognition practices to quantify the synergy of material and symbolic motivation; (2) Performance Evaluation Criteria, integrating assessment orientation and metric design to measure the institutionalization of NM practices; (3) Leadership Support, merging advocacy behavior and modeling by example to assess the strength of managerial value signaling; (4) Resource Allocation, linking financial and infrastructure support to reflect the provision of tangible resources; and (5) Organizational Culture, fusing humanistic atmosphere and narrative orientation to describe the depth of NM-related value integration. Together, these dimensions reflect both the material and symbolic aspects of institutional support that shape clinicians’ perceptions of legitimacy, feasibility, and organizational endorsement of NM practice.

#### 3.6.5 Perceived informal organizational support

The PIOS scale is designed to measure the multidimensional and dynamic support that clinicians receive for NM practice through informal social networks. Its development began with a literature review focusing on Bandura and Walters’s (55) observational learning mechanism and Wenger et al.’s (73) community of practice model. Grounded theory was then applied to analyze qualitative interview data, resulting in the identification of 10 first-level descriptive themes relevant to NM contexts: clinical observation (peer technique demonstration), experience emulation (peer case references), cross-institution interaction (academic network engagement), informal coordination (spontaneous task collaboration), situational communication (real-time problem-solving), daily interactions (informal experience exchange), instructional modeling (skills transfer in mentorship), interactive guidance (feedback in clinical teaching), social media inspiration (exposure to innovative ideas), and learning through online content (access to digital educational resources). Through axial coding, these were clustered into 5 sec-order dimensions: (1) Peer Modeling, which integrates clinical observation and experience emulation to reflect the value transmission of peer behaviors; (2) Spontaneous Collaboration, combining cross-institution interaction and informal coordination to assess the strength of non-hierarchical cooperation; (3) Contextual Sharing, merging situational communication and daily interactions to evaluate the accessibility of real-time support in informal settings; (4) Clinical Scaffolding, linking instructional modeling and interactive guidance to quantify the internalization of role-modeling behaviors; and (5) Media Influence, integrating social media inspiration and learning through online content to capture the catalytic role of digital resources in stimulating innovation. These dimensions provide a structured framework for understanding how informal, peer-driven, and media-enabled forms of support shape clinicians’ engagement with NM practice.

#### 3.6.6 Behavioral intention

In this study, the operationalization of Behavioral Intention (BI) follows both the classical TPB formulation and contemporary measurement guidelines. TPB defines BI as an individual’s subjective probability or willingness to perform a specific behavior in a given context Ajzen (27). The construct reflects a global motivational evaluation—capturing “whether” and “to what extent” an individual is willing to act—rather than a set of subcomponents that require decomposition. TPB treats BI as a global intention judgment, when the target behavior is clearly defined and context-specific, BI can be appropriately captured using a single, well-specified global item.

Consistent with this theoretical position, BI in this study is measured with a single 7-point Likert item (“I am willing to participate in NM practice”). This item simultaneously captures the three elements emphasized in TPB: behavioral specificity (“participate”), clarity of the behavioral target (“NM practice”), and voluntary commitment (“I am willing”). Empirical evidence supports the adequacy of single-item BI measures under such conditions. Wanous et al. (74) showed that when constructs are narrow and clearly defined, single-item measures demonstrate reliability comparable to multi-item scales (75). Further established that single-item measurement is appropriate when the construct is unambiguous and context-specific. In the clinical behavior domain, Hoeppner et al. (76) found that single-item BI measures can even outperform multi-item formats by reducing respondent burden and minimizing redundancy, thus improving predictive validity.

Given that the intention to engage in NM represents a unified motivational stance rather than a multidimensional behavioral performance, a single-item operationalization is both theoretically coherent and empirically justified for this construct. This approach also clearly distinguishes BI from practice behavior (PB), which, unlike BI, comprises multiple distinct behavioral components and thus requires a multidimensional measurement strategy (see Section 3.6.7).

#### 3.6.7 Practice behavior

The operationalization of PB in this study followed the standardized procedure of *theoretical framing → qualitative exploration and theme extraction → empirical refinement* (77), with the goal of constructing a measurement system that is comprehensive, clinically grounded, and conceptually coherent (see Section 3.4). Guided by the 22334 “Red-Flower Model” of core NM competencies—which has been incorporated into the Chinese National Expert Consensus on NM (78)—we conducted a rigorous thematic analysis of semi-structured interviews. This process yielded 16 first-level descriptive themes, representing concrete, observable components of NM practice: open-ended questioning, body language, echoing, summarizing and validating, appropriate silence, pacing and encouragement, acceptable language, inclusiveness, verbal information elicitation, non-verbal information elicitation, metaphor use, deconstruction, analysis, interpretation, creative skills, emotional description, expression of respect, expression of understanding, expression of support, and need responsiveness.

These 18 descriptive themes were analytically clustered into 4 sec-level conceptual dimensions, representing the core behavioral domains of NM practice:(1) Guidance, referring to the use of verbal and non-verbal techniques to actively facilitate narrative elicitation; (2) Listening, denoting the comprehensive capture of linguistic, paralinguistic, and non-verbal information; (3) Reflection, referring to interpretive and meaning-making processes that reconstruct patients’ narratives into clinically meaningful insights; and (4) Response, which captures respectful, supportive, and dignity-affirming clinician feedback.

Consistent with prior scholarship on the inherently multidimensional nature of NM practice behaviors (79–81), PB constitutes a multi-domain composite construct, where each domain plays a distinct and irreplaceable functional role—for instance, information gathering, narrative interpretation, emotional support, and relational attunement. Because these domains represent qualitatively different behavioral processes, a single-item measure cannot adequately represent the construct without producing an oversimplified or ambiguous assessment. By contrast, a multi-item measurement design ensures that each behavioral dimension is represented by several concrete, observable items, thereby achieving fine-grained behavioral capture. Aggregating multiple items within each dimension also substantially enhances psychometric reliability by reducing random measurement error. Even when respondents interpret an individual item with slight variation—an expected phenomenon given the complexity and wide behavioral range of narrative-based clinical skills—the composite multi-item score provides a more stable and accurate estimate of the respondent’s true behavioral tendencies. This psychometric robustness is essential for capturing the nuanced, context-dependent nature of NM practice behaviors.

#### 3.6.8 General information

The demographic section of this study amassed data encompassing participants’ gender, age, marital status, educational background, monthly income, professional title, department, nature of the organization, and years of experience.

### 3.7 Data analyses

To rigorously test the extended theoretical model grounded in TPB, this study adopted a two-stage hybrid analytic strategy integrating SEM and ANN. This approach was designed to capitalize on the strengths of both techniques—SEM enables theory-driven causal inference and hypothesis testing, while ANN compensates for its limitations in handling non-linear relationships and high-order variable interactions, offering enhanced predictive accuracy. This dual-stage methodology combines the interpretive strength of SEM with the predictive power of ANN, enabling a more nuanced understanding of the psychological and organizational mechanisms shaping physicians’ NM practices. It further enhances the extended TPB model by capturing both the linear and non-linear components of decision-making within complex clinical environments. This sequential integration is not merely methodological convenience, but reflects an epistemological complementarity—where SEM tests deductive, theory-driven paths, and ANN explores non-linear interactions and high-order patterns among theory-supported predictors that may remain obscured in linear models. All statistical analyses were conducted using SPSS 26.0 and AMOS 26.0.

In the first stage, SEM was employed to validate both the measurement and structural models, examining the hypothesized effects of ATT, SN, PBC, POS, and PIOS on physicians’ intention to practice NM. Prior to estimation, we assessed univariate normality through skewness and kurtosis coefficients, and reported descriptive statistics (means and standard deviations) for all core variables. Maximum Likelihood Estimation (MLE) was used to estimate parameters, given its robustness and suitability for large-sample structural modeling (82). Model fit was evaluated using conventional indices, ensuring both statistical rigor and theoretical adequacy.

In the second stage, ANN was introduced to further model the complex, non-linear interrelationships among key predictors identified by SEM. ANN is a biologically inspired computational architecture composed of interconnected nodes capable of simulating the neural activity of the human brain (83, 84). Through a feedforward–backpropagation learning mechanism, the ANN iteratively adjusts its internal weights to minimize prediction error, allowing it to capture latent patterns and high-order interactions that SEM—restricted to linear paths—may overlook (34, 35, 85). However, ANN’s “black-box” nature prevents it from offering clear parameter estimates or formal hypothesis testing, thus limiting its suitability for theory development and policy targeting. To address this, the study adopted a sequential SEM–ANN design: SEM was first used to confirm theoretically grounded relationships and identify significant predictors; these validated predictors were then entered into the ANN as input nodes to determine their relative importance in predicting behavioral intention.

Furthermore, to elucidate the combinatorial patterns of psychological constructs underlying BI, this study employed K-means clustering analysis using variables identified by SEM-ANN as input indicators for characteristic profiling. The optimal cluster number (K) was determined through concurrent validation via the Elbow Method and Silhouette Coefficient. Post-clustering, ANN-predicted behavioral intention scores (BI_Predicted) were integrated to develop a Psychological Motivation-Behavioral Intention Coupling Profiling Model. This model delineates distinct behavioral archetypes among healthcare professionals by coupling heterogeneous psychological variable combinations with intention levels. Additionally, contingency tables and chi-square tests were implemented to examine distributional heterogeneity of the derived profiles across organizational dimensions—including hospital level, regional development level, and clinical department type.

## 4 Results

### 4.1 Measurement model testing

To ensure measurement quality, a series of reliability and validity assessments were conducted. Internal consistency was first evaluated using Cronbach’*α*. As reported in Table 3, all constructs demonstrated excellent internal consistency, with Cronbach’s α coefficients ranging from 0.915 (Listening/Reflection) to 0.960 (POS), exceeding the conventional threshold of 0.80. A confirmatory factor analysis (CFA) was subsequently performed to examine the measurement model. All standardized factor loadings exceeded 0.775, indicating strong item-construct relationships (86). The composite reliability (CR) values ranged from 0.918 to 0.961, surpassing the recommended minimum of 0.70 (87), while the average variance extracted (AVE) values varied between 0.709 and 0.858, exceeding the standard 0.50 benchmark (88). Together, these results confirm strong convergent validity of the scales.

**Table 3:** Measurement model evaluation (*N* = 855).

Construct	Dimension	Measurement items	Load^a^	Cronbach’α	CR	AVE
Attitude (ATT)		ATT1	0.891	0.946	0.948	0.858
		ATT2	0.963			
		ATT3	0.922			
Subjective Norm (SN)		SN1	0.944	0.929	0.933	0.779
		SN2	0.947			
		SN3	0.912			
		SN4	0.699			
Perceived Behavioral Control (PBC)		PBC1	0.924	0.946	0.946	0.854
		PBC2	0.913			
		PBC3	0.936			
Perceived Organizational Support (POS)		POS1	0.921	0.960	0.961	0.832
		POS2	0.927			
		POS3	0.878			
		POS4	0.941			
		POS5	0.890			
Perceived Informal Organizational Support (PIOS)		PIOS1	0.852	0.944	0.945	0.776
		PIOS2	0.887			
		PIOS3	0.842			
		PIOS4	0.891			
		PIOS5	0.930			
Practice Behavior (PB)	Guidance(G)	G1	0.805	0.923	0.924	0.709
		G2	0.845			
		G3	0.887			
		G4	0.814			
		G5	0.826			
	Listening(L)	L1	0.832	0.915	0.918	0.737
		L2	0.869			
		L3	0.875			
		L4	0.829			
	Reflection(RF)	RF1	0.855	0.915	0.922	0.747
		RF2	0.902			
		RF3	0.889			
		RF4	0.783			
	Response(RS)	RS1	0.775	0.932	0.935	0.744
		RS2	0.908			
		RS3	0.933			
		RS4	0.834			
		RS5	0.828			

CR, Composite reliability; AVE, average variance extracted.

Discriminant validity was assessed using the Heterotrait-Monotrait Ratio (HTMT), a robust and conservative approach. As shown in Table 4, all HTMT values ranged between 0.310 and 0.844, remaining below the accepted threshold of 0.85 (89). These findings indicate satisfactory discriminant validity and support the psychometric soundness of the measurement model.

**Table 4:** HTMT ratio.

Construct	F1	F2	F3	F4	F5	F6	F7	F8	F9
ATT	—								
G	0.455	—							
L	0.478	0.817	—						
RF	0.454	0.806	0.844	—					
RS	0.526	0.728	0.824	0.826	—				
PBC	0.455	0.633	0.673	0.684	0.662	—			
SN	0.427	0.533	0.598	0.564	0.585	0.565	—		
POS	0.310	0.409	0.412	0.450	0.388	0.531	0.656	—	
PIOS	0.341	0.446	0.480	0.516	0.464	0.574	0.585	0.637	—

ATT, attitude; G, guidance; L, listening; RF, reflection; RS, response; PBC, perceived behavioral control; SN, subjective norm; POS, perceived organizational support; PIOS, perceived informal organizational support; PB, practice behavior.

### 4.2 Structural model and testing of hypotheses

To evaluate the structural relationships and test the proposed hypotheses, the initial structural model was estimated using the MLE method (90). To assess multivariate normality, the standardized multivariate kurtosis coefficient (C. R. value) was calculated. Following Bentler (91) criterion, a C. R. value exceeding 5.00 is considered indicative of severe non-normality. In this study, the C. R. value reached 1100.991, clearly suggesting significant deviations from multivariate normality. To account for this violation and ensure the robustness of parameter estimation, the Bollen–Stine bootstrap procedure was applied. This technique adjusts the model’s chi-square statistic and associated *p*-value to reduce bias induced by non-normal data distributions (92).

Model fit was subsequently evaluated using multiple goodness-of-fit indices. As shown in Table 5, the modified model demonstrated excellent fit across all indicators, indicating that the structural model provided a strong and statistically valid representation of the observed data.

**Table 5:** Fitting results of the structural equation model.

Fitting index	Fitting standard	Model	
		Initial model	Modified model
Chi-square freedom ratio *χ*^2^/df	1 < *χ*^2^/df < 3 Good	8.219	1.576
Root mean square error of approximation, RMSEA (90%CI)	<0.05 Good	0.092	0.026
Goodness of fit index, GFI	>0.90 Good	0.646	0.967
Adjusted goodness of fit index, AGFI	>0.90 Good	0.606	0.963
Normed fit index, NFI	>0.90 Good	0.827	0.967
Confirmatory fit index, CFI	>0.90 Good	0.845	0.988
Bentler and Bonett’s nonnormed fir index, TLI	>0.90 Good	0.836	0.987

Utilizing SEM, the hypothesized relationships within the proposed model were validated. Specifically, the study examined six relationships, ascertaining the direct effects of ATT, PBC, SN, POS and PIOS, as well as the direct influence strength and significance of BI on PB. The findings indicate that, among hypotheses H1 to H6, only H5 was not supported, while the remaining hypotheses were all statistically significant at the *α* = 0.05 level, as shown in Table 6. ATT, SN, PBC, and PIOS all demonstrated a positive impact on doctors’ NM behavioral intentions, whereas it was unexpected that the study did not confirm a significant effect of POS on BI. BI has a positive influence on PB.

**Table 6:** Hypotheses testing results.

Paths	Relationships	Standardized path coefficients	*p*-value	Confidence Interval	Result
H1	ATT → BI	0.116	***	0.044 ~ 0.187	Supported
H2	SN → BI	0.118	**	0.036 ~ 0.198	Supported
H3	PBC → BI	0.335	***	0.258 ~ 0.413	Supported
H4	PIOS→BI	0.399	***	0.311 ~ 0.482	Supported
H5	POS → BI	0.015	n.s.	−0.070 ~ 0.103	Not supported
H6	BI→PB	0.47	***	0.414 ~ 0.518	Supported

*p < 0.05; **p < 0.01;***p < 0.001; n.s., not significant. ATT, attitude; G, guidance; L, listening; RF, reflection; RS, response; PBC, perceived behavioral control; SN, subjective norm; POS, perceived organizational support; PIOS, perceived informal organizational support; BI, behavioral intention; PB, practice behavior.

### 4.3 Outputs of neural network analysis

In this study, we employed a Multilayer Perceptron (MLP) architecture with a feed-forward backpropagation learning algorithm to evaluate the (93) importance of the predictors identified in the SEM analysis. The MLP model was selected due to its strong representational capacity—particularly its ability to capture complex nonlinear relationships—its structural flexibility, and its well-established training procedures, which make it one of the most frequently used neural network techniques in behavioral research (84, 93–95). Consistent with prior studies applying ANN to behavioral prediction (96–99), this study adopts an MLP architecture to capture nonlinear and interaction effects that SEM alone may not fully model.

The architecture of the neural network was configured as follows. The number of input neurons was determined by the number of predictor variables included in each model, while the output layer contained a single neuron corresponding to the dependent variable. To balance theoretical parsimony and model capacity, and in line with best practices in the field, the ANN adopted a single-hidden-layer structure (100–102). As there is no universally accepted heuristic for determining the optimal number of hidden neurons, this parameter was estimated through SPSS’s automated trial-and-error optimization procedure (103, 104). Given its suitability for modeling nonlinear patterns, the Sigmoid activation function was used in both the hidden and output layers (96, 99).

To reduce the risk of overfitting and ensure robust generalization performance, several strategies were implemented. The dataset was first divided into a 90% training set and a 10% holdout testing set. During model development, a systematic 10-fold cross-validation procedure was applied to the training portion (96, 97, 99, 105). This was complemented by monitoring of the validation error during the training process, which further prevented the model from memorizing idiosyncratic patterns in the training data.

**Figure 2:**
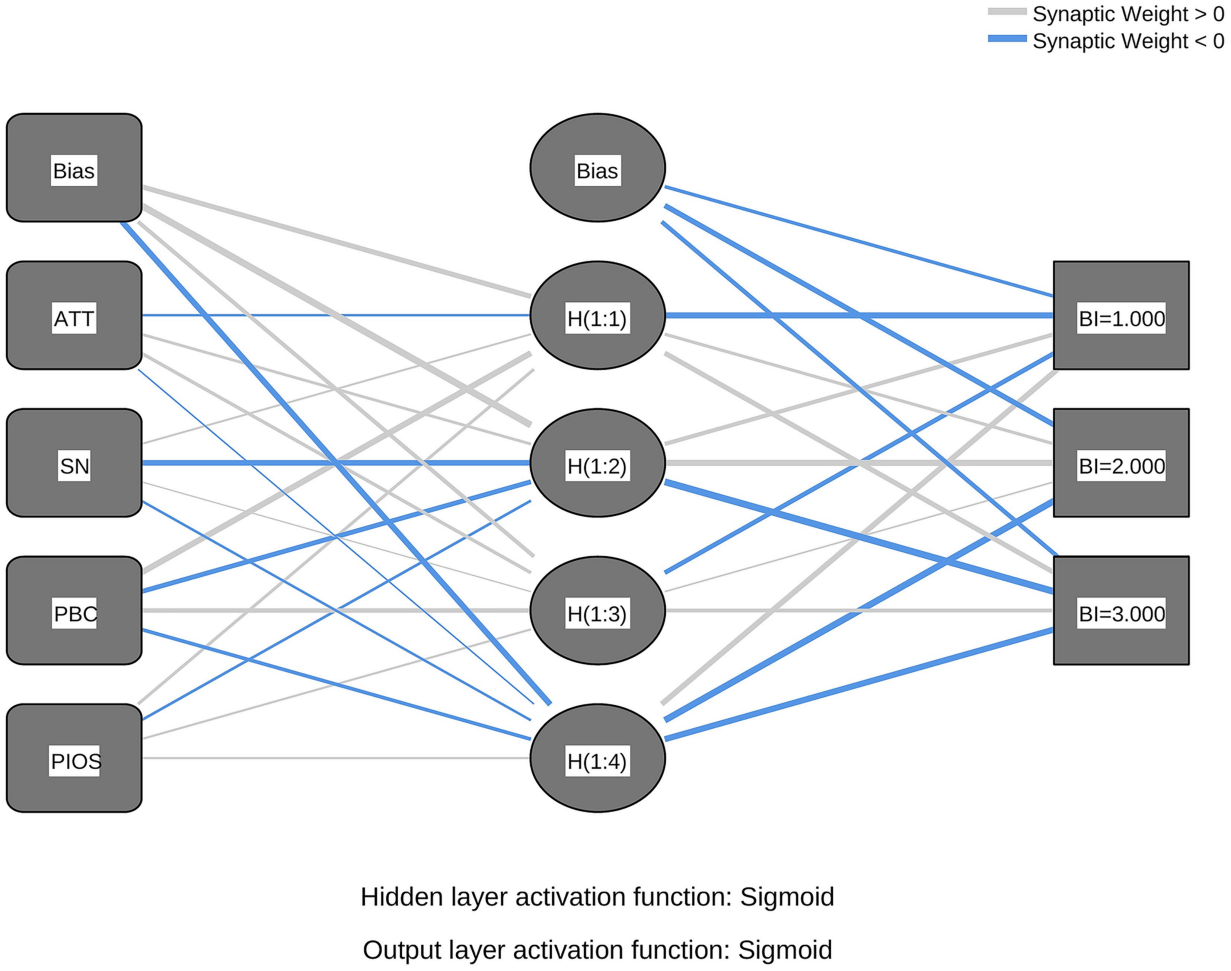
ANN model A. A multilayer perceptron network diagram with input variables (ATT, SN, PBC, PIOS) and Bias connected to hidden layer nodes (H1:1–H1:4). Hidden and output layer activation functions are sigmoid. Synaptic weights are marked as positive or negative.

**Figure 3:**
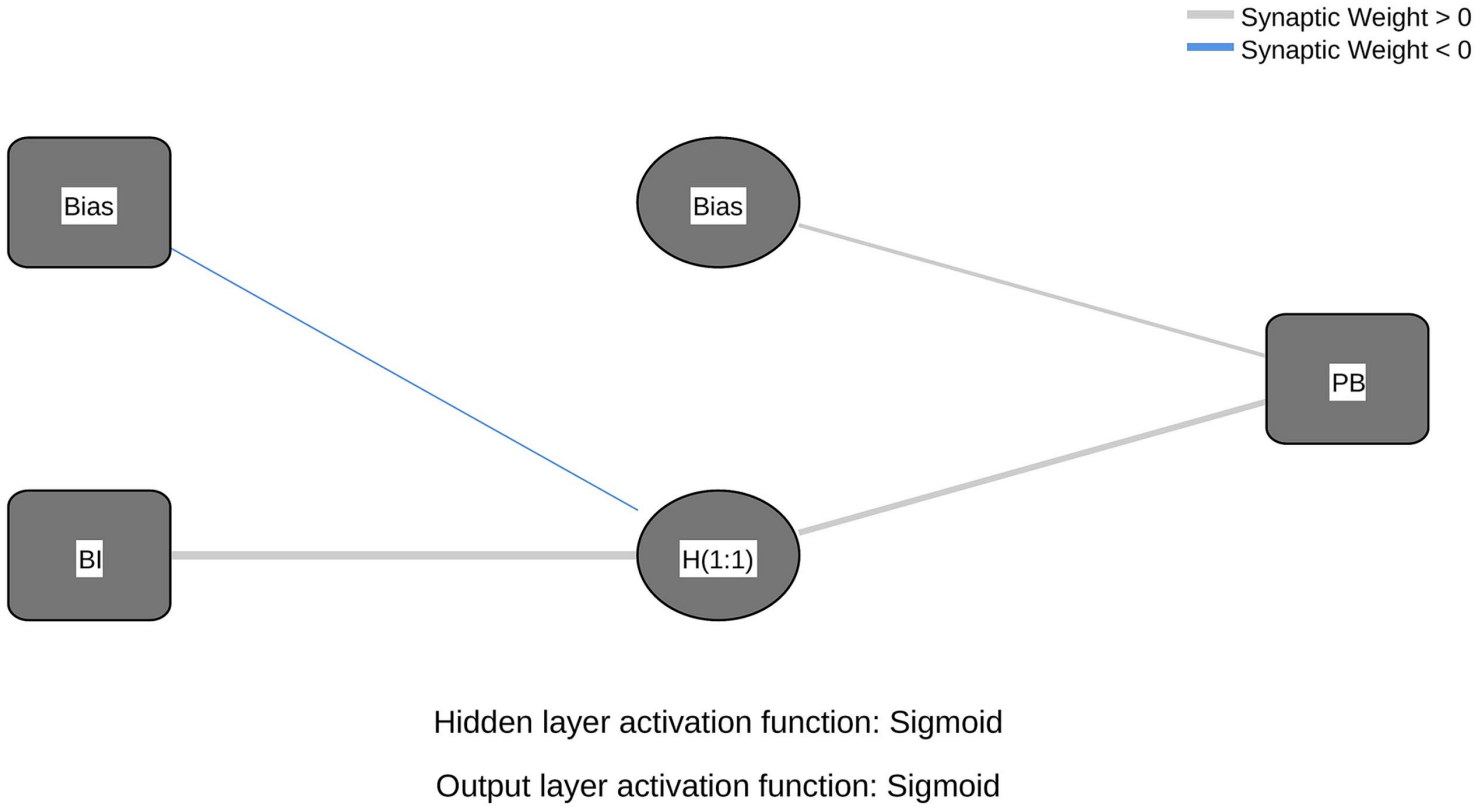
ANN model B. A multilayer perceptron network diagram showing input variables (BI) and Bias connected to hidden nodes and then to the output(PB). Activation functions are sigmoid, with positive and negative synaptic weights indicated.

Based on the significant predictors identified in the preceding SEM analysis, two ANN models were constructed. Model A was developed to predict BI and included four neurons in the input layer (ATT, SN, PBC, and PIOS) and one output neuron representing BI. Model B examined the determinants of PB, with BI as the single input neuron and PB as the output neuron. The architectures of both models are presented in Figures 2, 3.

Model performance was rigorously assessed using the root mean square error (RMSE) and sum of squared errors (SSE) across all cross-validation folds and the independent test set. As summarized in Table 7, both models achieved strong predictive accuracy. Model A yielded average RMSE values of 0.427 (training) and 0.421 (testing), while Model B achieved higher precision, with RMSE values of 0.079 (training) and 0.075 (testing). The low standard deviations of these indicators across folds indicate consistent and stable model performance (96–98).

**Table 7:** SSE and RMSE values for training and testing.

Neural Network	Model A				Model B			
	SSE (Training)	SSE (Testing)	RMSE (Training)	RMSE (Testing)	SSE (Training)	SSE (Testing)	RMSE (Training)	RMSE (Testing)
1	138.803	14.944	0.423	0.435	4.718	0.524	0.079	0.075
2	136.873	15.937	0.424	0.410	4.783	0.437	0.079	0.074
3	141.332	13.938	0.428	0.412	4.832	0.388	0.079	0.069
4	136.383	13.199	0.422	0.385	4.816	0.443	0.078	0.078
5	158.597	14.347	0.454	0.406	4.858	0.409	0.079	0.070
6	139.455	17.046	0.427	0.435	4.592	0.643	0.077	0.087
7	133.783	15.965	0.417	0.431	4.857	0.389	0.079	0.069
8	139.342	21.467	0.427	0.488	4.747	0.825	0.079	0.094
9	144.966	12.874	0.431	0.414	5.046	0.351	0.081	0.061
10	136.002	12.063	0.419	0.391	4.813	0.423	0.079	0.070
Mean	140.554	15.178	0.427	0.421	4.806	0.483	0.079	0.075
SD	6.700	2.558	0.010	0.028	0.110	0.139	0.001	0.009

RMSE, Root mean square of errors; SSE, Sum of square error; SD, Standard.

To further evaluate predictive capability, the ANN-based R^2^ statistic was calculated using the following formula (97, 106) (Equation 1):


$$R^{2}=1-\frac{\text{RMSE}}{S_{y}^{2}}$$ (1)

$S_{y}^{2}$ represents the variance of the preferred output as indicated by the mean (SSE) of the testing process. The outcome suggests that the ANN model predicts 97.2% of BI. For the SEM model, predictive power was assessed using the squared multiple correlation coefficient (*R*^2^), which indicates the proportion of variance explained in the dependent variable. The *R*^2^ value of BI was 22.1% for the SEM analysis as opposed to that of the ANN analysis which disclosed an *R*^2^ value of 97.2%. This contrast highlights the added value of ANN in capturing complex, nonlinear relationships that may be underestimated in traditional SEM frameworks. Together, these findings support the use of a hybrid SEM–ANN approach to maximize both theoretical interpretability and predictive performance.

Finally, a sensitivity analysis was conducted for Model A to quantify the relative contribution of each input variable to BI. Importance values were computed by perturbing each input and measuring the resulting changes in network predictions. As shown in Table 8, PBC emerged as the strongest predictor, followed by PIOS, SN, and ATT. Table 9 summarizes the differences in predictor importance rankings between the SEM and ANN models, suggesting that ANN is capable of modeling complex and potentially non-additive interactions among predictors that linear path models may not fully capture.

**Table 8:** Normalized variable importance.

Predictors	Normalized importance
ATT	40%
SN	45%
PBC	100%
PIOS	91%

ATT, Attitude; SN, Subjective norm; PBC, Perceived behavioral control; PIOS, Perceived informal organizational support.

**Table 9:** Comparison between SEM and ANN results.

Predictors	SEM		ANN	
	Standardized PATH coefficients	Ranking	Relative importance	Ranking
ATT	0.097	4	0.381	4
SN	0.108	3	0.423	3
PBC	0.337	2	0.949	1
PIOS	0.380	1	0.868	2

ATT, Attitude; SN, Subjective norm; PBC, Perceived behavioral control; PIOS, Perceived informal organizational support.

### 4.4 Behavioral intention profiles based on TPB predictor combinations

**Figure 4:**
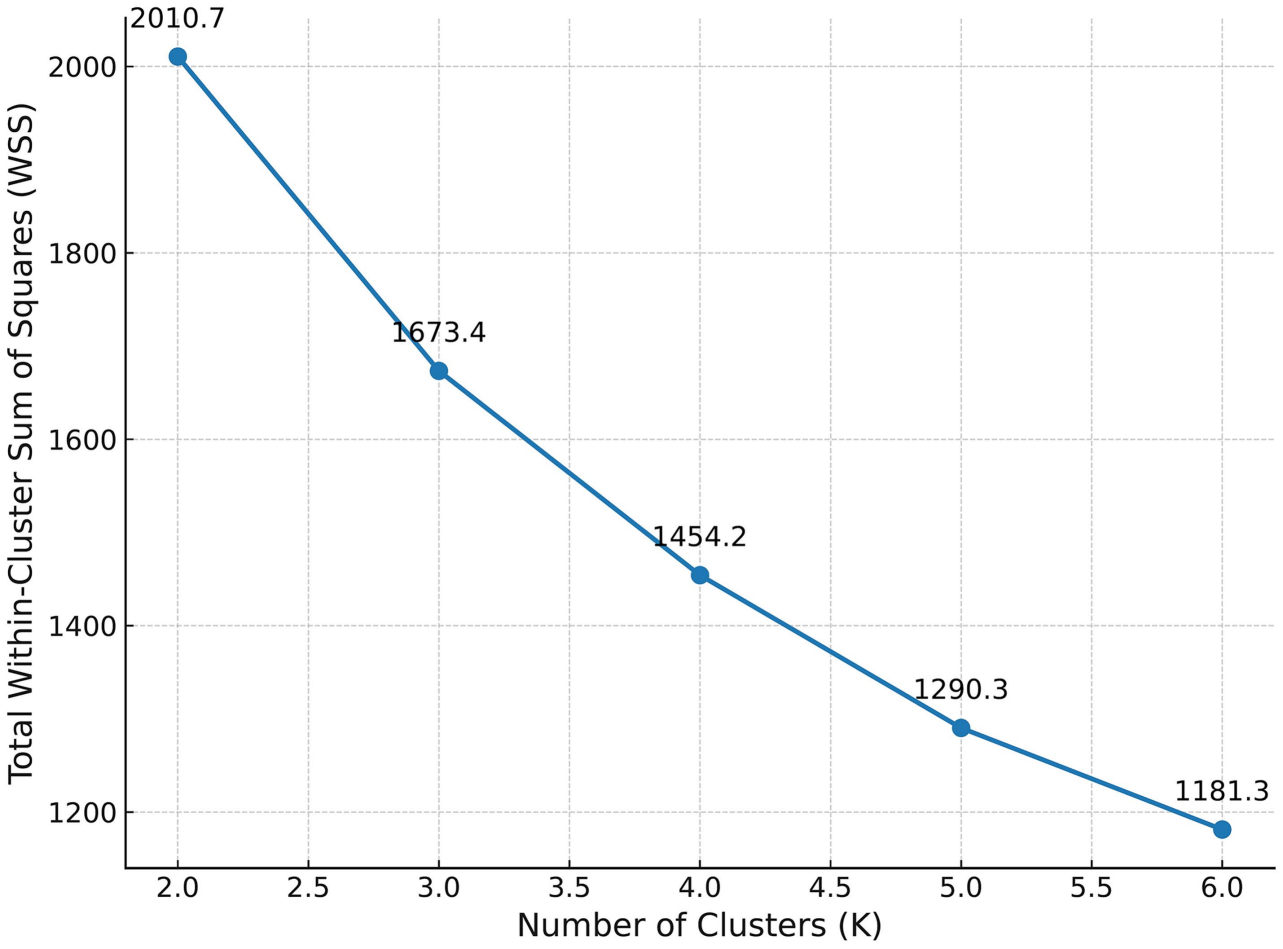
Elbow method for determining optimal K. A line chart plotting the total within-cluster sum of squares (WSS) against the number of clusters (K) ranging from 2 to 6. The curve decreases as K increases, with WSS values annotated from 2010.7 to 1181.3, illustrating the elbow method for cluster selection.

Based on the SEM-ANN results, four key variables—ATT, SN, PBC, and POS—were selected as input indicators for K-means clustering analysis. All four variables were measured using 7-point interval scales, with consistent metric properties and no missing data. Prior to clustering, all inputs were standardized to meet the sensitivity requirements of K-means regarding Euclidean distance. To determine the optimal number of clusters (K), both the Elbow Method and the Silhouette Coefficient were employed to systematically evaluate solutions ranging from K = 2 to K = 6. The within-cluster sum of squares (WSS) showed a steep decline from K = 2 to K = 4, indicating substantial improvement in intra-cluster compactness. After K = 4, the marginal decrease in WSS leveled off, and the silhouette coefficient reached its peak at 0.49, suggesting that K = 4 achieves the best balance between within-group cohesion and between-group separation. Further inspection of marginal explanatory gains for K = 5 and K = 6 revealed that the added interpretability from additional clusters dropped below 9%, signaling a risk of over-segmentation. Taken together, considerations of compactness, separation, and model parsimony support the selection of K = 4 as the optimal solution (see Figure 4).

The four-cluster solution derived from K-means analysis was interpreted as representing distinct psychological–motivational profiles rather than mere statistical groupings. This interpretation is grounded in a multi-layered justification. First, the clustering variables (ATT, SN, PBC, PIOS) were theoretically selected based on their established predictive validity within the extended TPB framework, as confirmed by prior SEM and ANN analyses. Second, profile interpretation was based on their configurational patterns and their systematic relationship with the BI-predicted scores, which varied coherently across clusters—from the lowest BI in the “Attitudinally Compliant, Structurally Suppressed” profile to the highest in the “Fully Aligned, High-Potential Implementers” profile. Finally, the external validity of these profiles was evidenced by their systematic and clinically meaningful distribution across different clinical settings. Together, this theory-driven, criterion-linked, and contextually patterned evidence supports treating the clusters as meaningful archetypes for understanding differential clinician engagement pathways with NM.

**Figure 5:**
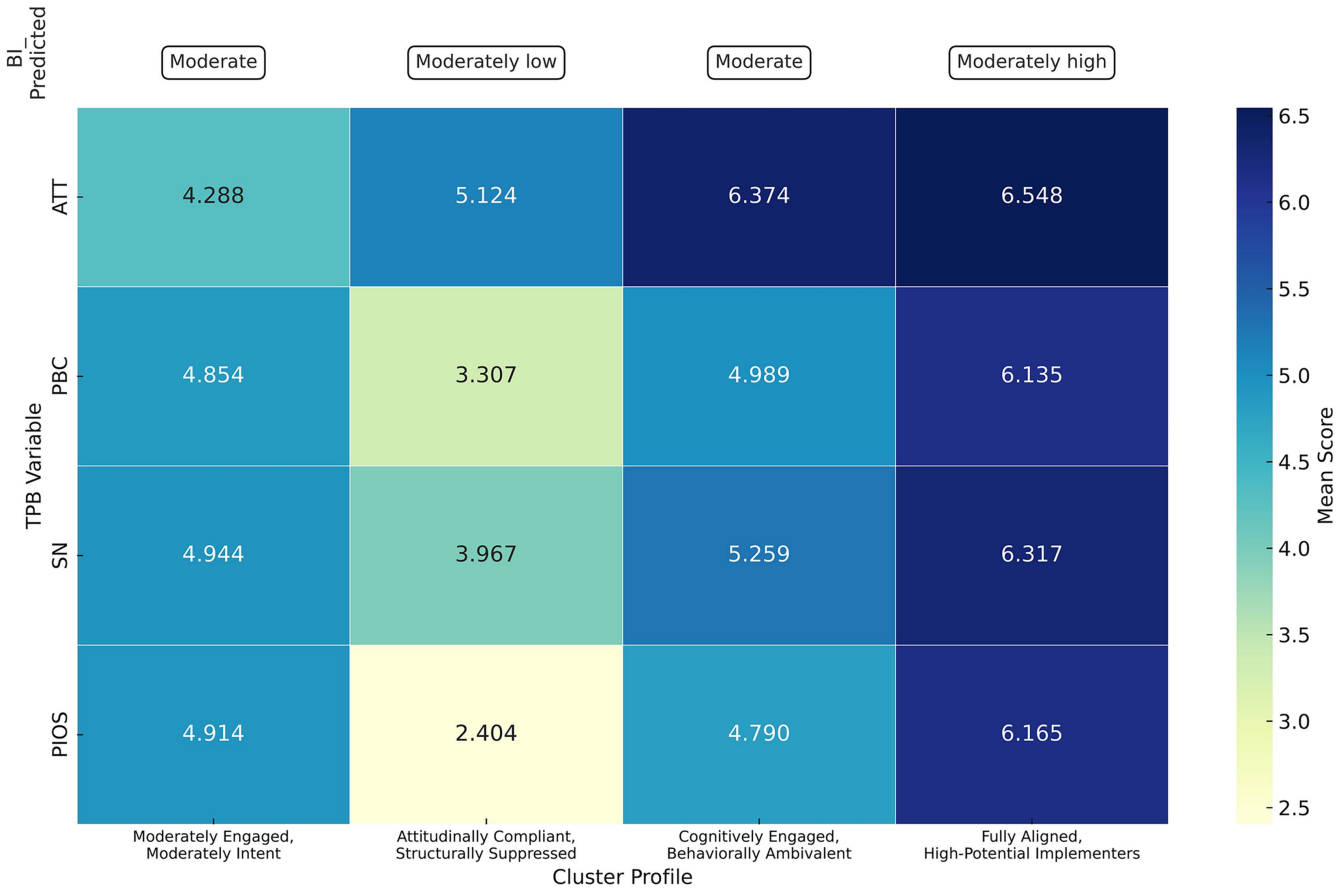
Cluster profiles of clinicians by TPB variables and BI_Predicted. A clustered bar and profile plot presenting four cluster groups: “Moderately Engaged, Moderately Intent,” “Attitudinally Compliant, Structurally Suppressed,” “Cognitively Engaged, Behaviorally Ambivalent,” and “Fully Aligned, High-Potential Implementers.” The clusters are compared across ATT, PBC, SN, and PIOS variables, with mean scores ranging from 2.4 to 6.5.

Building upon the previous K-means clustering results (K = 4), this study further analyzed cluster characteristics by incorporating the ANN-predicted Behavioral Intention (BI_Predicted) scores to determine the behavioral inclination of each group. Given that behavioral intention may exhibit nonlinear threshold shifts, a categorical interpretation of BI_Predicted was retained, consistent with the stage-based nature of cognitive-to-behavioral transformation. This classification resulted in the identification of four clinician types, each representing a unique motivational profile and corresponding level of intention to engage in NM (shown in Figure 5).

Cluster 1: moderately engaged, moderately intent.

This cluster displays a generally moderate motivational profile across all key variables (ATT = 4.288, PBC = 4.854, SN = 4.944, PIOS = 4.914), suggesting a psychologically balanced yet underactivated structure. Clinicians in this group may recognize the conceptual value of NM but exhibit insufficient momentum to transform that recognition into actionable intention. Their behavioral intention remains moderate, reflecting a state of motivational neutrality rather than resistance. This group may not yet perceive NM as urgent, normative, or feasible enough to prioritize in daily clinical routines.

Cluster 2: attitudinally compliant, structurally suppressed.

Clinicians in this cluster report a moderately high attitude toward NM (ATT = 5.124), yet their scores on subjective norm (SN = 3.967), perceived behavioral control (PBC = 3.307), and perceived organizational implementation support (PIOS = 2.404) are the lowest across all clusters. Their predicted behavioral intention is also the lowest, reflecting a significant motivational deficit. This profile suggests that while these clinicians intellectually acknowledge the value of NM, they operate within environments that provide limited normative endorsement, structural facilitation, or peer reinforcement. The resulting intention–motivation mismatch points to an unmet need for institutional alignment and support to translate belief into action.

Cluster 3: cognitively engaged, behaviorally ambivalent.

This cluster displays a strong motivational profile in attitude (ATT = 6.374) and subjective norm (SN = 5.259), indicating high cognitive endorsement of NM. However, scores for perceived behavioral control (PBC = 4.989) and perceived organizational implementation support (PIOS = 4.790) are comparatively moderate, and behavioral intention does not rise proportionally, remaining at a middling level. This pattern reflects a classic intention–action gap, where strong belief does not readily translate into decisive behavioral commitment, possibly due to perceived feasibility constraints or insufficient reinforcement.

Cluster 4: fully aligned, high-potential implementers.

This cluster demonstrates near-ceiling scores across all TPB dimensions—attitude (ATT = 6.548), perceived behavioral control (PBC = 6.135), subjective norm (SN = 6.317), and perceived organizational implementation support (PIOS = 6.165)—indicating a fully aligned motivational structure. Although their behavioral intention is moderately high rather than maximal, the convergence of internal conviction and external support suggests a high level of readiness for NM engagement. These clinicians may be well-positioned to act as early adopters, particularly if enabling organizational conditions are maintained.

To further explore how motivational-intentional profiles are distributed across organizational dimensions, we examined three structural variables: geographic region, hospital tier, and clinical department (see Table 10). First, no significant regional differences were observed in the distribution of cluster types across areas with varying levels of socioeconomic development (χ^2^ = 3.141, *p* = 0.791), suggesting that clinicians’ motivation–intention configurations are not strongly shaped by regional macroeconomic disparities. Second, cluster distribution varied significantly across hospital tiers (*χ*^2^ = 22.297, *p* = 0.001). Cluster 2 (Attitudinally Compliant, Structurally Suppressed) was more prevalent in provincial-level hospitals, while Cluster 4 (Fully Aligned, High-Potential Implementers) dominated in municipal-level hospitals. Cluster 3 (Cognitively Engaged, Moderately Intent) had the highest proportion in county-level hospitals. This pattern suggests that the structural positioning and resource allocation of institutions may shape both motivational alignment and behavioral intention. Third, a significant difference was also found across clinical specialties (χ^2^ = 26.240, *p* = 0.036). Cluster 2 was most prominent among physicians in emergency and pediatrics. Cluster 1 was most common in internal medicine and obstetrics/gynecology, whereas Cluster 4 was predominant in surgical departments.

**Table 10:** Distribution of TPB-based motivational profiles across region, hospital level, and clinical department.

Contextual variable	Categorization	Cluster 1	Cluster 2	Cluster 3	Cluster 4	*χ* ^2^	*p*
regions	Affluent regions	33 (34.0%)	13 (35.1%)	105 (39.5%)	158 (34.7%)	3.141	0.791
	Moderately affluent areas	48 (49.5%)	17 (45.9%)	128 (48.1%)	232 (51.0%)		
	Less affluent regions	16 (16.5%)	7 (18.9%)	33 (12.4%)	65 (14.3%)		
Hospital level	Provincial-level hospitals	48 (49.5%)	20 (54.1%)	100 (37.6%)	180 (39.6%)	22.297	0.001
	Municipal-level hospitals	45 (46.4%)	12 (32.4%)	120 (45.1%)	233 (51.2%)		
	County-level hospitals	4 (4.1%)	5 (13.5%)	46 (17.3%)	42 (9.2%)		
Clinical department	Emergency and critical care medicine	2 (2.1%)	4 (10.8%)	21 (7.9%)	38 (8.4%)	26.240	0.036
	Internal medicine	48 (49.5%)	8 (21.6%)	91 (34.2%)	153 (33.6%)		
	Surgery	19 (19.6%)	7 (18.9%)	54 (20.3%)	100 (22.0%)		
	Pediatrics	3 (3.1%)	5 (13.5%)	10 (3.8%)	14 (3.1%)		
	Obstetrics and gynecology	5 (5.2%)	1 (2.7%)	12 (4.5%)	18 (4.0%)		
	Others	20 (20.6%)	12 (32.4%)	78 (29.3%)	132 (29.0%)		

## 5 Discussion

This study aimed to develop a comprehensive model to analyze the predictors of doctors’ intention and behavior regarding NM practice. By incorporating PIOS and adopting the ANN-SEM method, we constructed a more explanatory model. Through hypothesis validation and training/testing the ANN model, several key findings were obtained.

PBC emerged as the strongest predictor of physicians’ intention to engage in NM, aligning with prior research that highlights PBC as a critical determinant of practice adoption among healthcare professionals (107, 108). While some TPB-based studies in health domains identify attitude or subjective norm as the dominant factor, the prominence of PBC in this study underscores its unique salience in the context of NM—a practice that requires clinicians to manage time-intensive, emotionally demanding, and communicatively complex interactions. PBC reflects physicians’ self-assessed capacity to execute a behavior, encompassing perceived feasibility and self-efficacy. In high-pressure clinical environments, where NM implementation entails significant opportunity costs, PBC becomes particularly consequential. Clinicians with stronger PBC are more likely to sustain engagement in the face of uncertainty, structural rigidity, and competing demands (109). This finding reinforces the theoretical proposition that behavioral intentions are not only shaped by motivational alignment, but also by an individual’s confidence in their ability to overcome systemic and procedural constraints. In the case of NM, where short-term incentives are limited and institutional routines often discourage interpretive flexibility, PBC becomes a gatekeeper for volitional translation. It highlights the need for not only normative promotion, but also capacity-building strategies to enhance clinicians’ behavioral control in real-world settings (110).

In addition to PBC, both ATT and SN significantly and positively influenced clinicians’ BI. This finding aligns with existing evidence highlighting the critical roles of affective evaluations and social influences in shaping health professionals’ decision-making processes. For instance, Alradini et al. (111) emphasized SN’s substantial impact in clinical contexts where medical practices are heavily influenced by patient and societal expectations. Similarly, Deng and Liu (112) underscored the pronounced effects of social norms and attitudinal factors on clinicians’ BI. Notably, the stronger influence of SN compared to ATT may reflect China’s collectivist cultural orientation and the unique socio-professional dynamics of medical practice. Within collectivist cultural contexts, societal expectations (e.g., from patients, peers, and institutions) often supersede individual attitudes in guiding behavioral choices (113, 114). This normative hierarchy becomes particularly pronounced in clinical environments, where clinicians typically prioritize meeting patient, peer, and organizational expectations when making behavioral decisions (115).

Contrary to theoretical expectations (116–118), this study found that POS did not significantly predict clinicians’ intention to engage in NM. This result does not imply that POS is ineffective; rather, it reflects the contextual boundary conditions under which organizational support can exert motivational influence—conditions that are not fully satisfied within the dual structural characteristics of China’s public healthcare system, namely bureaucratic hierarchy and chronic resource constraints.

According to organizational support theory, the motivational effect of POS depends on its structural visibility and degree of institutional embeddedness (119). For a practice such as NM, which requires considerable emotional labor, time investment, and cognitive effort, clinicians’ trust in organizational support depends heavily on the credibility and continuity of formal commitments (16). Although many hospitals express policy-level support for NM, rigid resource allocation mechanisms and saturated clinical workloads often prevent such support from becoming operational. As a result, POS frequently takes a **s**ymbolic rather than substantive form—for instance, NM is not included in performance evaluations, nor are there dedicated time allowances or integrated Electronic Medical Record tools—creating a misalignment between organizational endorsement and practical enablement (52, 53).

Moreover, organizational resources in Chinese clinical settings are highly diluted. High patient volumes and heavy clinical responsibilities limit clinicians’ capacity to absorb additional requirements associated with NM. Training and equipment support offered by hospitals tend to be diffused across competing clinical tasks and thus fail to offset the perceived costs of NM practice. Limited managerial engagement and prioritization of rigid clinical demands further weaken the credibility of POS, reducing its influence on frontline decision-making (18–20).

Prior research also indicates that when organizational support fails to translate into consistent practice conditions, trust mechanisms are undermined, diminishing the predictive power of POS (52, 53). Thus, the nonsignificant effect of POS in this study likely reflects contextual constraints rather than a theoretical contradiction.

Further supporting this line of reasoning, the cluster analysis points to a possible structural misfit contributing to POS’s weakened influence: the misalignment between standardized organizational incentives and the heterogeneous realities of clinical practice. The data reveal significant variation in physicians’ intentions to adopt NM across hospital tiers and departmental settings, indicating that motivational orientations are shaped by localized institutional and operational conditions rather than uniformly distributed across the system. This pattern highlights a critical institutional limitation in China’s current NM implementation framework. Organizational support mechanisms—such as performance assessments and training programs—are predominantly standardized in form. Yet, in the case of NM, which inherently requires interpretive communication, active listening, and sustained emotional engagement, such uniform structures often lack the temporal flexibility and organizational scaffolding necessary for practice-level adoption (120). The diminished predictive effect of POS observed in this study, therefore, likely reflects the insufficient contextual responsiveness and structural alignment with the differentiated demands of clinical environments.

While not originally part of the TPB framework, PIOS emerged in this study as the second strongest predictor of behavioral intention, pointing to the limitations of TPB’s individually centered assumptions. This finding supports ongoing efforts to extend the model by incorporating contextual and structural determinants that shape intention enactment (31). This interpretation aligns with prior evidence suggesting that medical humanities implementation, including NM, is highly context-sensitive—particularly in environments that promote guided reflection and peer-based learning (121–123). The explanatory value of PIOS may lie in its role in activating informal organizational processes that facilitate the translation of narrative concepts into clinical practice. First, informal learning settings such as Balint groups and peer case-sharing may offer reflective spaces that enhance clinicians’ emotional attunement, procedural fluency, and professional identification with NM (124). Compared to formal structures, informal organizations emphasize interpersonal interaction and individualized support, fostering stronger engagement with NM practices (113, 125). Second, NM relies heavily on tacit knowledge—embedded in emotional attunement, experiential narratives, and co-construction with patients—which is often transmitted through socialized practices such as clinical storytelling and communities of practice. These mechanisms are positively associated with innovative work behaviors and adaptive expertise (126–129). The elevated social interactivity inherent in informal organizations may facilitate both emotional resonance and tacit knowledge exchange across clinicians, peers, and patients. This dual reinforcement pathway may, in turn, support professional identity integration and help explain the enhanced behavioral intention observed among physicians perceiving strong organizational support.

Moreover, the prominent influence of PIOS observed in this study aligns closely with the collectivist orientation and relational culture characteristic of Chinese healthcare institutions (130). Within hospital departments—the primary clinical microsystems—peer expectations, shared norms, and day-to-day social interactions often exert a more immediate and compelling impact on clinicians’ behavioral choices than distant administrative directives. Such informal, relationally grounded social structures provide clinicians with contextualized, credible, and actionable cues about whether a new practice is valued and feasible.

When NM is endorsed by respected peers or integrated into existing professional communities, its adoption becomes socially normative, emotionally resonant, and personally meaningful. In contrast, formal organizational support within large, hierarchical public hospitals may sometimes be perceived as aspirational rather than reliably translated into sustained operational resources. As a result, clinicians may regard formal POS as symbolically important but insufficient to shape everyday practice without complementary peer-level reinforcement.

In this context, PIOS functions as a critical “reality check” and translational mechanism. Support from trusted colleagues and mentors offers authentic, experience-based evidence that NM is both valuable and practicable, helping clinicians overcome initial skepticism or feasibility concerns that formal top-down policies alone may not fully address. Thus, the stronger predictive effect of PIOS does not diminish the theoretical significance of organizational context; rather, it refines our understanding of how support is activated in practice. It suggests that in collectivist, high-pressure clinical environments, social learning, interpersonal validation, and peer modeling may serve as more direct and effective levers for motivating the adoption of complex, relationally intensive practices such as NM.

The typology emerging from the TPB-based clustering offers more than descriptive segmentation—it reveals latent tensions, structural asymmetries, and psychological discontinuities that shape clinicians’ engagement with NM. Rather than existing along a linear spectrum of intention strength, the four profiles suggest qualitatively distinct motivational architectures, each shaped by the convergence—or fracture—of attitudinal, normative, control, and contextual influences. Cluster 1 underscores the phenomenon of cognitive inertia: moderate alignment across TPB dimensions fails to catalyze behavioral readiness, implying that conceptual endorsement alone is insufficient without salience or urgency. In contrast, Cluster 2 represents a structurally disenfranchised group—clinicians who intellectually accept NM yet operate within a void of institutional reinforcement. Here, behavioral inaction is not the product of resistance, but of environmental desynchronization: when attitudes are unsupported by normative cues, control perception, or embedded organizational signals, motivation decays into disuse. Cluster 3 exemplifies a more subtle paralysis: despite deep cognitive engagement, ambivalence persists, likely due to an imbalance between internal motivation and contextual legitimacy. This “intention fatigue” marks the limits of internal conviction when unsupported by feasible pathways to enactment. Only Cluster 4 demonstrates motivational coherence across levels, where internal and external forces converge to create psychological traction. Yet even in this high-potential group, behavioral intention remains cautiously tempered—suggesting that in emotionally demanding practices like NM, even well-aligned structures must be activated through credible opportunities and sustained cultural validation. Taken together, these findings extend TPB beyond individual volition, calling attention to how intention is not merely generated, but contextually nurtured, constrained, or eroded within multi-level practice ecologies.

Building on the typological analysis, this study theoretically advances TPB across three interrelated dimensions, with significant implications for health psychology. First, it empirically validates the predictive utility of PIOS, proposing a theoretically grounded extension that formally integrates institutional support into behavioral intention modeling. Second, the research demonstrates that intention formation is not a simple linear additive process but requires higher motivational coherence—where the synergy of ATT, SN, and structural dimensions proves more critical than isolated reinforcement of any single dimension. Third, it reveals how structural asymmetries and fragmented organizational support weaken the behavioral translation of individual commitment, challenging the classical assumption that “cognitive endorsement naturally leads to behavioral intentions” (131–133). These findings hold particular relevance in high-stakes clinical environments—characterized by emotionally taxing labor, operational overload, and intense resource competition—where physicians’ behavioral mobilization depends critically on a synergistic ecosystem of institutional, cultural, and psychological support. Our work does not reject TPB’s core cognitive logic but advances its ecological reconstruction, preserving its cognitive foundation while emphasizing the activation (and dysregulation) of multilevel support systems in practice. This perspective aligns with health psychology’s emerging paradigm shift: understanding individual motivation as an outcome of systemic, multifactorial interactions. Within this context, our study not only strengthens TPB’s explanatory power in medical behavior but also provides pivotal scaffolding for developing context-sensitive intention-modeling frameworks.

Building on the preceding theoretical insights, this study further investigates how motivational clusters are distributed across different hospital tiers. The stratified distribution of motivational profiles across hospital tiers reveals how institutional environments do not merely allocate resources but actively shape the motivational ecology underlying clinicians’ engagement with NM. In provincial-level hospitals, the dominance of structurally suppressed profiles (Cluster 2) underscores a paradox: even in settings endowed with advanced expertise and infrastructure, rigid procedural norms and hierarchical governance may constrain agency, limiting the translation of professional values into volitional intent. This suggests a form of institutional over-specialization, where high-capacity systems may inadvertently disincentivize non-instrumental innovations such as NM (134). By contrast, municipal hospitals appear to offer a more permissive implementation climate (Cluster 4), marked by policy openness and organizational adaptability. Here, the alignment of internal and external enablers supports a more coherent motivational trajectory, positioning these institutions as fertile ground for NM mainstreaming (135). Meanwhile, county-level hospitals illustrate a different constraint pattern (Cluster 3): strong cognitive endorsement exists, but intention stalls due to structural fatigue—characterized by limited incentives, inadequate staffing, and lack of practice infrastructure (136). Together, these patterns suggest that motivational formation is deeply embedded in local institutional logics, and that the viability of NM adoption hinges less on attitude alone than on the system’s capacity to scaffold intention with credible pathways for enactment.

Building on the previously discussed tier-based institutional synergies, this study further analyzed the distribution of motivational clusters across clinical departments, revealing a more nuanced pattern of heterogeneity. In emergency and pediatric departments, clinicians were predominantly categorized into Cluster 2, indicating a pattern of structural inhibition despite attitudinal endorsement. Although these professionals generally affirm the conceptual value of NM, their behavioral intention remains critically low—primarily due to attenuated PBC and limited PIOS. Prior research has shown that in emergency medicine, intensive workload, shift-based fatigue, legal uncertainty, and emotional depletion diminish clinicians’ sense of psychological safety and agency (137–139). Pediatric care, meanwhile, is often mediated through triadic communication involving caregivers, adding emotional and cognitive strain that complicates narrative engagement (140, 141). Collectively, these constraints create a “frozen motivation” dynamic, wherein cognitive acceptance fails to translate into behavioral readiness due to pervasive contextual misalignment. Second, in internal medicine and obstetrics-gynecology departments, clinicians are predominantly clustered in Cluster 1. Physicians in internal medicine, although routinely engaged in chronic disease management and complex patient communication—contexts conducive to narrative sensitivity—often work under fragmented schedules and time-pressured consultations (142, 143). Obstetricians and gynecologists similarly acknowledge the emotional relevance of NM but operate under heightened medico-legal scrutiny, which incentivizes protocolized, defensive communication patterns (144, 145). This risk-averse orientation reduces narrative flexibility, leading to an implicit mechanism of “conceptual endorsement but behavioral avoidance”. In contrast, surgical departments showed the highest concentration of Cluster 4, reflecting motivational coherence and strong behavioral intention. This challenges conventional perceptions of surgery as purely technocratic and efficiency-driven. The growing emphasis on perioperative communication, patient-centered recovery, and collaborative team workflows has fostered greater narrative receptivity among surgeons (146, 147). Additionally, stable routines, role clarity, and cohesive team structures contribute to elevated PBC and PIOS, together facilitating a psychologically enabled and intention-rich environment conducive to NM implementation.

The findings of this study extend beyond theoretical contributions, offering an evidence-based roadmap for integrating NM into practice. The affective and relational logic of PIOS is particularly instructive for formal organizations. Identifying distinct motivation-intention profiles enables a shift from uniform to stratified interventions. Hospital managers can use these profiles to allocate resources effectively. For “Structurally Inhibited” clinicians (Cluster 2), priority must be given to removing environmental barriers (e.g., workflow optimization) while connecting them with “Fully Synergistic” peers (Cluster 4) via formal mentoring—a strategy leveraging PIOS’s relational essence to foster collective effort and belonging. For groups with moderate or ambivalent engagement (Clusters 1 and 3), the key is combining PIOS’s cultural permeation with targeted formal support. This involves micro-training in discrete narrative skills to build confidence and cultivating communities of practice for peer-based internalization of NM values—a form of soft power beyond mere institutional incentives. Moreover, the “Fully Synergistic” group (Cluster 4) is a strategic resource. Empowering them as advocates and mentors institutionalizes PIOS’s informal support, making NM competence visible and reinforcing the subjective norm that “narrative matters,” thereby aligning formal systems with informal culture.

The conclusions of this study offer transferable insights at the mechanistic level, with both empirical support and defined contextual boundaries. Pecifically, within the extended TPB framework (ATT, SN, PBC) and contextual support variables, our study highlights how clinicians’ intention to adopt NM is shaped, and which factors are most likely to function as critical levers in high-pressure clinical environments. In particular, our findings suggest that in Chinese clinical culture—where peer modeling, social learning, and informal learning networks are commonly observed—PIOS carries more direct explanatory value and clearer intervention implications for promoting complex and relational practices such as NM. By contrast, the exact path coefficients and effect sizes may vary across regions.

We systematically included diverse areas across Zhejiang based on economic development, healthcare resource accessibility, and geographic characteristics (including developed urban areas and relatively underdeveloped or mountainous regions). We also covered different hospital levels (provincial, municipal, and county), forming a “dual-stratified” sample structure to capture key structural variations commonly present in China’s public healthcare system. Therefore, what our study supports most strongly for transfer is the portability of the relational structure and underlying mechanisms: key mechanisms such as PIOS remained evident even when substantial contextual variation was already present within the sample, increasing their explanatory potential for settings with similar institutional structures and clinical pressure conditions.

Cross-regional application should be based on contextual similarity. Specifically, when the target region/hospital resembles our sample in key conditions—for example, similar levels of workload and patient-volume pressure; active intra-department peer interaction and informal learning networks; and NM-related practices that rely primarily on peer modeling and community-based exchange—then the mechanism-level conclusions identified in our study (especially the joint effects of TPB cognitive factors and informal support such as PIOS on intention and behavior) are more likely to be informative. In contrast, because path coefficients and effect sizes may shift with regional differences in resource endowment, policy environments, and organizational culture, we provide only a brief caution that parameter-level results should not be generalized mechanically and should be further validated and calibrated through replication studies in other provincial samples.

At the policy level, these findings call for a strategic shift from principled endorsement of medical humanities toward actively constructing a supportive ecosystem. Cultivating informal social structures related to PIOS should become a key dimension of policy design. Policymakers are well-positioned to implement systemic changes that address the core motivational barriers identified in this study: integrating narrative competence into national physician licensing and continuing education standards to grant it formal legitimacy; piloting value-based payment models and reforming hospital performance evaluation frameworks to include humanistic indicators, thereby aligning formal systems with NM principles. Crucially, given the contextual heterogeneity revealed by the cluster analysis, national strategies should invest not only in developing tailored NM implementation toolkits but also—and more importantly—in proactively nurturing PIOS as a critical resource by funding cross-institutional learning networks and peer mentoring programs. Such policy-level impetus provides fertile ground for informal support to grow, allowing the strengths of PIOS—rooted in emotional connection and shared experience—to flourish and sustain the social architecture needed for lasting practice change. This ecological approach, characterized by “formal systems as the foundation, empowered by informal culture,” encapsulates the core policy implication of learning from PIOS and serves as the fundamental guarantee for translating NM from concept into sustained practice.

This study has several limitations, which also point to valuable directions for future research. First, the cross-sectional design collected data on behavioral intention at only a single point in time. Future studies could adopt longitudinal or experimental designs to dynamically track changes in behavioral intention and actual behavior, thereby providing stronger supporting evidence. Second, self-reported data may be subject to inherent social desirability and recall biases; however, anonymous surveys with retained identity codes helped mitigate these effects. Third, the data were primarily collected from Zhejiang Province. Although stratified sampling across different economic levels and hospital tiers within the province was employed to enhance sample heterogeneity, caution is warranted when extrapolating the conclusions to other regions with significant differences, as effect sizes may be influenced by geographical factors. Future research could validate the model in broader regions and examine the moderating effects of contextual factors such as local policies and resource accessibility, thereby further clarifying the model’s boundary conditions and generalizability.

## 6 Conclusion

This study extends the TPB framework to identify key predictors of physicians’ intention to practice NM, revealing that ATT, SN, PBC, and PIOS collectively shape behavioral intention, with PBC emerging as the strongest predictor. Through the innovative application of a hybrid ANN-SEM method, the research successfully captured complex nonlinear relationships among variables, thereby enhancing the model’s predictive accuracy. Importantly, K-means clustering analysis delineated four distinct motivation-intention profiles—termed “Moderately Engaged, Moderately Intent,” “Attitudinally Compliant, Structurally Suppressed,” “Cognitively Engaged, Behaviorally Ambivalent,” and “Fully Aligned, High-Potential Implementers”—whose distribution demonstrated significant organizational heterogeneity, with particularly instructive patterns observed between emergency/pediatrics and surgical departments. Theoretically, by integrating PIOS as a key contextual variable, the findings advance TPB by conceptualizing intention formation as a systemically coupled process driven by the synergy of ATT, SN, and PBC, moving beyond a linear additive model. Practically, this work provides an evidence-based, data-driven framework for healthcare managers and policymakers to design stratified and context-sensitive implementation strategies. Matching interventions to specific clinician profiles and fostering a supportive policy ecosystem can accelerate the systematic integration of NM, ultimately contributing to a more humane, reflective, and effective healthcare culture.

## Data Availability

The original contributions presented in the study are included in the article/Supplementary material, further inquiries can be directed to the corresponding author.
