# Supplementary material for: Determinants of Chinese physicians’ engagement in narrative medicine: a comprehensive SEM-ANN analysis
Source: Front Med (Lausanne). 2026 Jan 15;12:1694846. doi: 10.3389/fmed.2025.1694846 (PMC12851974; doi:10.3389/fmed.2025.1694846)
Supplement: Supplementary file 1 [file Table_1.docx]

| First-level Descriptive Theme | Sceond-level Analytic Theme | Third-level Analytic Theme | Frequency | Original data example |
| --- | --- | --- | --- | --- |
| Attitude | Value Cognition | People-oriented | 38 | D80 I think a good doctor must have medical ethics. The core of narrative medicine is people-oriented, being good to patients, having empathy, being able to feel the difficulties of patients and their families, otherwise, it's impossible to write patient stories and do narrative medicine well. |
|  |  | Medical Humanistic Care |  | D101 The doctor's own view on narrative medicine is very important. For example, if I subjectively don't agree with narrative medicine, don't think it can have a good effect on medical humanistic care, then this thing definitely can't be carried out well. So I think to carry out narrative medicine, first of all, we need to reach a consensus, if we all recognize its valuable contribution in humanistic care, think this thing is really reliable, then we can do it together well. |
|  | Tool Evaluation | Practicality | 56 | D8 Certainly, narrative medicine has developed quite well, whether it's in theory or practice, it's now widely recognized in the medical community. The key is that it's a tool that can truly implement humanistic care in clinical settings, showing that this is entirely feasible in practice. |
|  |  | Acceptability |  | D74 Narrative medicine is a good thing, doctors can accept it, at least that's how I feel. It's really a great thing. Being able to write out one's thoughts and ideas, diagnostic and treatment approaches, so that everyone can understand, and also to let the general public know about our work. There is just one thing, this is quite time-consuming, writing, revising, summarizing patient conditions all require a lot of time, and our workload is already large, so it might be a bit difficult in practice, but that doesn't affect my view that it's good. |
|  | Confidence | Development Potential | 26 | D3 Currently, the general trend is gradually placing more emphasis on medical humanities. In the future, it will most likely become an essential skill for physicians. Not only are young doctors willing to learn it, but many hospitals may also gradually incorporate it into their teaching and assessment systems.Once everyone gets the hang of it, and the supporting support is in place, it won't just be something a few people are doing, but a natural part of clinical diagnosis and treatment. |
|  |  | Future Prospects |  | D77 There's a medical reform going on now, and in the future, the public's recognition of the doctor-patient relationship will definitely increase, and interactions will become more harmonious. I think, riding this wave, the development of narrative medicine will definitely be good, and its implementation will be smoother. |
| Subjective Norm | Policy Norm | Policy Advocacy | 23 | D22 I think the practice of narrative medicine relies on policy advocacy. First, there need to be encouraging policies for us practitioners, and then we need to raise social awareness of narrative medicine through policy guidance, so that everyone can accept this model. D36 To carry out narrative medicine, policy advocacy is definitely necessary. We need to start with policy-making, and the leaders as policymakers need to first understand this area of knowledge in order to formulate appropriate rules. Society also needs to rely on policy to promote and educate this model, and our medical personnel need corresponding professional training support. D26 The practice of narrative medicine cannot be separated from policy advocacy. The policy environment needs to increase its promotion and publicity. D30 To properly carry out the practice of narrative medicine, and to let others understand that we are helping them, publicity is definitely necessary, and this also needs to be advocated and guided at the policy level. |
|  |  | Institutional Support | 12 | D36 In fact, for us medical personnel, to carry out narrative medicine, first understanding and learning more is one aspect, but more crucially, there need to be corresponding institutional provisions to promote and disseminate it. This is how it can truly be implemented. I think medical personnel generally aren't uneducated, they have a strong sense of social responsibility, and even a sense of mission. The key lies in whether there are supporting standardized procedures and macro-level systems within the industry and society to underpin our efforts in this matter. |
|  | Organizational Norm | Management Promotion | 17 | D11 We still need the organization to endorse this. When carrying out the practice, we also need to clarify the scenarios where it is mandatory. Diseases like tumors and chronic illnesses are probably quite suitable. Then we should discuss how to conduct it in compliance with regulations. All these are aspects that organizational management needs to consider. I heard that some hospitals in southern China have done a great job in this regard. They specially set up narrative sharing centers and carry out this work in an organized way from top to bottom. Such solid support really motivates people a lot. |
|  |  | Practice Encouragement | 34 | D66 I believe that narrative medicine practice needs to be genuinely encouraged. First of all, there needs to be corresponding support and cooperation. If it produces results, some recognition and preference could be given, which would also reflect its value. If the feedback after implementation is positive, it could be fully promoted as a highlight of the hospital. We could also organize regular gatherings for patients who have received help from narrative medicine to share their experiences. In addition, the hospital could host more related activities to motivate everyone to engage more enthusiastically. |
|  | Education Norm | Curriculum Integration | 43 | D35 I believe that narrative medicine practice needs to start with educational institutions, and it is necessary to open specialized courses. Now, narrative medicine has been included in the standardized training, and only by cultivating well can we ensure that doctors always maintain their original aspiration for medicine. A2 It is essential to add courses related to narrative medicine in schools, and now that standardized training has included it, we should also organize special lectures to help doctors master this ability earlier.  D22 For narrative medicine to be implemented, it cannot do without the support of courses in educational institutions. Now that standardized training has included this content, if we also pair it with daily skills training and psychology-related further education, we will find it easier to carry out. |
|  |  | Teaching Innovation | 16 | D34 In education, we can have more diverse approaches. For outpatient doctors, we can organize some relevant learning activities, and we can also go out for exchanges and learning, such as learning from psychological counselors, acquiring related knowledge across fields, and allowing everyone to master this aspect of skills, which would be more practical.  D13 To say that narrative medicine can be implemented, the methods of education need to be adjusted. When I was studying medicine, my mind was full of hard courses like anatomy and physiology, and there was no teaching of narrative medicine related to humanities. I had to slowly figure it out in my work later. You young people are really lucky; medical courses are becoming broader and broader. I suggest that narrative medicine can be arranged from the time medical students enter school, starting with elective courses to adapt, and gradually turning them into required courses, just like how I taught clinical oncology at the university, from elective to required, gradually advancing. Moreover, not only the courses for medical students need to be adjusted, but also the continuing education in a doctor's career needs to be optimized, and even the physician promotion examination can include some narrative medicine content, which can assess professional ability and also make everyone pay more attention, making subsequent implementation smoother. |
|  | Patient Norm | Communication Needs | 35 | D106 In fact, patients' minds need doctors to practice narrative medicine, which can just meet their needs for recognition and good communication. Imagine, if through narrative medicine, patients feel their conditions are valued and affirmed by doctors, their emotions will definitely improve, and their enthusiasm and initiative for subsequent treatment will be higher. The communication and treatment actions that follow will be smoother. Anyone who gets such attention and communication will be more willing to cooperate. D4 I really think narrative medicine should be widely promoted. When patients seek medical treatment, they actually need this way of communication, which fits their communication needs. Practicing this can help with patients' compliance during medical treatment. After all, when communication is in place, patients will be more willing to follow the doctor's plan. D111 Patients need doctors to practice narrative medicine, which can meet their needs for good communication with doctors and promote a good doctor-patient relationship, making communication smoother for both parties. |
|  |  | Humanistic Expectations | 34 | D8 In the medical environment, because it has always been an accurate situation where doctors and patients, official doctors and patients should always be in a state of inequality, it's not just about the inequality at the knowledge level. Because when we tell patients about treatment plans, options a, b, c, three or four types, they might, uh, still feel that even if you explain it to them in more common language, they might still feel it's wrong. So I think if we let patients tell their whole disease story like a story, we can arouse their supply, or we can understand their disease with a state of empathy, then I think we can gain some new communication effects. D26 The need for knowledge dissemination, I also see some doctors' lectures, often their clinical diagnosis and treatment ideas are not consistent. There is a need for some review or some restrictions. It can mislead patients and increase communication difficulties. D105 I think first of all, the current doctor-patient relationship, especially on the internet, some have made the doctor-patient relationship a bit worse. |
| Perceived Behavioral Control | Ability Cognition | Practical Ability | 57 | D14 Specific abilities are very important. First of all, clinical expertise must be solid, the foundation must be strong; then, communication must be worked on, such as how to explain the condition and feelings to patients in simple terms, with more affinity, so that patients are willing to open up and actively cooperate with treatment. These practical communication skills are quite important. AH6 First, you have to thoroughly understand and read the core logic and methodology of narrative medicine, know how to guide patients and how to listen; then, start with the patients around you, try to practice more, accumulate experience, and with practice, you will gradually develop these specific operational skills, and be able to carry out narrative medicine well. |
|  |  | Technical Mastery | 48 | D62 For instance, parallel medical records are different from clinical records. They require precise documentation of the patient's unspoken emotions, concerns, and life background, and these details need to be correlated with the illness and treatment plan. After writing, the ability to use these records is crucial, reviewing them to check for any missed core demands and to consider how to improve communication next time. This is a technical task and it's time-consuming. |
|  | Problem-Solving Efficacy | Adaptability | 39 | D106 does indeed have some patients who need more empathetic communication, but it's not realistic for every patient to be the same. Consider our current medical environment, where we have to see 80 patients in a morning, which is very different from foreign doctors who see only 5 patients in the same time frame. This requires us to be flexible, adjusting our communication style and depth according to the patient's personality, the severity of their condition, and the time constraints. We can't use a fixed approach; only by doing so can we implement narrative medicine effectively. |
|  |  | Analytical Ability | 36 | D1 needs to be able to analyze situations. For example, when we receive patients, we will understand their family and social situations, as well as their symptoms. This includes regular communication with family members after the patient is admitted to the ward, to meet the patient's reasonable demands.  D7 doctors need to analyze the regional characteristics and the public's level of understanding to find the appropriate communication method. For instance, in this area, the public's knowledge level is relatively backward, making communication more difficult.  D62 When patients are narrating, they may not be very coherent and may express their own emotions or understanding of the disease. At this point, I need to find the key information from these scattered narratives, discard the excess emotions, and accurately determine what the patient needs. Only in this way can narrative medicine be done well. |
|  | Self-efficacy | Confidence Level | 55 | D74 When I first started, I always felt uncertain, afraid of not guiding the patient well, and afraid of not empathizing enough. I didn't dare to delve into the patient's inner thoughts. Later, I tried to do it gradually and found that patients are actually quite willing to say more. My confidence grew slowly. Now I dare to do more. If I don't have confidence, it's hard to do this. |
|  |  | Motivation Maintenance | 31 | D80 When the outpatient service gets busy, seeing dozens of patients in a morning, sometimes I really want to save some effort, just asking about the illness and prescribing medicine would be enough, without the heart to talk about the patient's inner matters. Sometimes, when I encounter patients who are unwilling to say much, or after talking for a long time without any progress, I feel a bit discouraged and just want to give up. But every time I think that some patients are willing to cooperate with treatment because I talked a bit more, or they say to me 'Doctor, after you said that, I feel much better', I feel that this is worth doing. It's really with this thought of wanting to help patients, and the sense of achievement after seeing the results, that I can keep going. |
| Perceived Organizational Support | Incentive Mechanism | Reward Measures | 48 | D2 work benefits, salary incentives, these are all necessary. The unit should be able to provide them because narrative medicine does require you to spend energy on it. With these practical guarantees and incentives, people will have the motivation.  D60 If narrative medicine can be recognized, and the organization can give more salary and bonuses as material rewards, then everyone will definitely be willing to execute it seriously. |
|  |  | Recognition System | 21 | D105 should engage in narrative medicine, and there should be a relevant system in place. It's not about seeking extra benefits; it's about having the effort and hard work one puts in being acknowledged. For instance, more consideration could be given during merit-based evaluations, and leaders and units could provide clear affirmation. Such recognition encourages people to work diligently. |
|  | Performance Appraisal | Assessment Orientation | 31 | D2 To advance narrative medicine, the organization needs to provide clear assessment guidelines. To be honest, it's quite difficult to measure the time and effort doctors invest in this field. Without a clear direction for assessment, it's hard for hospitals to incentivize in other ways.  D8 I think narrative medicine should be assessed, but it's a bit of a conundrum to do so. It's not easy to assess, and I wonder how other hospitals handle it? If it's included in the assessment, patient satisfaction and the evaluation of the entire treatment process could be used as references. |
|  |  | Indicator Design | 23 | D121 Which indicators should be used, preferably quantitative ones. I think the time spent with each patient during consultation should definitely be considered; time represents the most basic element of medical practice, without which it's impossible to proceed. Secondly, patient evaluations and profiles should also be examined. Only when doctors fully engage in narrative medicine can they create better profiles for patients. |
|  | Leadership Support | Advocacy Behavior | 19 | D35 Doctors should be encouraged more, especially from a leadership perspective, by saying more encouraging words. This might have a better effect. Because when we were children, we might have heard some words about how sacred doctors are, and then chose the medical profession. But later, when facing very heavy and complex clinical work, such sacred words are forgotten. If there's always a voice telling you how sacred doctors are, it might be very important and meaningful for the development of narrative medicine. |
|  |  | Example Modeling | 10 | D22 If leaders could provide some exemplary materials or share successful cases, that would serve as a great guiding force. Seeing a direction, people are more willing to follow and do a good job in narrative medicine. |
|  | Resource Allocation | Equipment Support | 32 | A10 has a language bank that includes various dialects as well as English, Japanese, Korean, etc. We have registered relevant personnel here, so that we can communicate smoothly with patients who have communication difficulties, which plays a significant guiding role in the development of narrative medicine.  D22 The setting up of places, the staffing, and the application and purchase of related facilities and equipment all require support from the unit. D2 I also think the work environment is necessary, because our current consultation environment is still quite public. For example, our hospital's psychiatric department has a dedicated consultation room, which is relatively better. But here, sometimes the environment can be quite noisy, and the office may not be particularly private. Sometimes, when it comes to something very private, patients may not feel comfortable speaking. If the hospital could provide a separate consultation room, a private space, patients would be more willing to share their inner thoughts, and narrative medicine could be carried out more smoothly. D20 Online efforts are also necessary, meaning we need to push in this direction. It's not just about promoting within the hospital and then nothing outside. We can use apps or mini-programs, and push relevant content through storytelling, allowing the public to indirectly access and understand, which can make subsequent clinical progress smoother. A3 Once the development starts, the consultation time and costs may both increase, and the hospital needs to increase support in this area. In addition, for patients with poor communication skills, it would be great if the unit could coordinate volunteers to help, this kind of human support is also effective. |
|  |  | Funding Support | 15 | D11 We may need to provide support to higher-level hospitals and increase the funding and support for our diagnosis and treatment. |
|  | Organizational Culture | Humanistic Atmosphere | 27 | D71 I think it's still about shaping the cultural atmosphere of narrative medicine. We can post patient thank-you letters and banners on the walls, so everyone can see them, and when the atmosphere gets better, everyone will want to participate. |
|  |  | Narrative Orientation | 20 | A1 Now we also emphasize this aspect. In fact, our hospital now advocates being patient-centered, which may make our vast medical staff fundamentally focus on this idea. |
| Perceived Informal Organizational Support | Peer Demonstration | Clinical Observation | 31 | A2 There is a role model demonstration, which is that when colleagues around us are doing well, I will see and learn from it, or the team atmosphere and team care are all needed. A3 It's also good to have someone who does well in this area, then leading everyone to do it together is better. There may need to be some records or meetings to communicate and exchange ideas. D9 When everyone pays so much attention to this, they will do it well. Sometimes it's about team collaboration, the team working together. For example, everyone can divide the work and collaborate, or have a role model start first, and then others follow and learn. |
|  |  | Experience Borrowing | 29 | D47 We still need to communicate more, talk more, and learn from the wrong answers. D18 During the weekly meetings, everyone will share cases, or share what they see during the diagnosis and treatment process, and also learn about the current more advanced knowledge and practices. |
|  | Spontaneous Collaboration | Cross-institutional Exchange | 22 | D109 I had previously gone to Peking Union Medical College for further studies, where I was able to access their practices and experiences in this field, which can be referenced. A11 At academic conferences, you'll meet colleagues from different hospitals and regions. We exchange cases, practices, and insights, which is a lot to learn from and helps us avoid detours. N4 Since there aren't many hospitals currently offering this, it was indeed difficult to get started. I attended a workshop where I learned directly, which is faster than figuring things out on my own. |
|  |  | Informal Coordination | 25 | D31 Sometimes we attend small lectures and talks in our daily lives. When people are willing to share, having this knowledge makes a big difference in how we perform. |
|  | Context Sharing | Scenario-based Communication | 30 | D19 We also discuss this patient's condition during our usual breaks, whether during lunch breaks or in the lounge. It's more like casual conversation, but this kind of communication is actually quite beneficial. It's considered casual chatting, but this kind of exchange is quite beneficial. We also talk about treatment guidelines, which I think is great for the progress of our team and department. |
|  |  | Daily Communication | 35 | D18 In terms of teamwork, information exchange and sharing are key. During our daily interactions, we share opinions and techniques, which is essentially sharing our knowledge base. D36 Colleagues should communicate more often when there's nothing pressing, learn more about each other's thoughts on patients, and share narrative and guiding techniques. We should learn from each other's strengths and good communication methods, then listen to patients' narratives. |
|  | Clinical Guidance | Demonstrative Teaching | 13 | D66 Just like in palliative care, the core is to understand the patient's personality and their views on the illness, then guide them accordingly. If the patient has a negative mindset, we need to help them confront possible outcomes, listen to what they most want to do and what they worry about the most, then do our best to help them resolve these issues, allowing them to peacefully go through their last journey and happily approach the end. In my outpatient clinic, I often see some teachers who will directly ask the elderly: 'If you really had to leave, what is the one thing you most want to do?' I often hear this question, and I learn from them. This approach allows patients to directly express their anxiety and worries, and we, as listeners, guide them and help them achieve these wishes. |
|  |  | Interactive Guidance | 12 | D121 Since narrative medicine is inherently a subjective humanistic system, beyond the basic elements of time and attitude, we can definitely create some typical cases. Have the junior students find some typical cases, see how they communicate with patients, express these processes in writing, and then have the supervising teachers review and analyze, pointing out inaccuracies in description and areas where communication could be improved. |
|  | Media Influence | Social Media Inspiration | 23 | A4 Online news and stories are also sources of learning, as are books. They all broaden our perspectives. Of course, if there's an opportunity, inviting experts to give lectures is also a good way to expand knowledge. |
|  |  | Online Content Learning | 17 | D36 We can usually learn the standardized knowledge in our field on online platforms like science popularization websites. The content learned online is both professional and easy to understand, making it very convenient to use when communicating with patients and their families. Moreover, we can also learn about which experts and peers are doing well in this field through online resources, and share this information and experience with patients and their families. In this way, communication is both substantial and well-founded, which can really help to improve narrative medicine. |
| Narrative Medicine Practice Behavior | Guiding | Open-ended Questioning | 92 | D115 Generally speaking, we used to say that we should not induce patients. But with some patients, you still need to appropriately give them some questions, otherwise they won't tell you everything. Doctor from Taizhou City Tumor Hospital-Doctor 3: Most doctors will definitely ask more questions, such as what discomfort you first felt, and how did it develop? When we ask, they will gradually clarify the situation. D13 This means we are still guiding, your questions guide the patient in which direction to answer you. For example, if we focus on sleep issues, we focus on whether they wake up early or have trouble sleeping, so when prescribing sleep or sedative drugs, we will focus on promoting patients' rapid sleep or extending their sleep time, so we will adjust based on this. D22 What doctors should do is because some patients may not be willing to talk much, they need questions to guide, and then get some clues for treatment or diagnosis. Basically, it's open-ended questions first, and then within the scope of open-ended questions, and then targeted questions, so that the questions are comprehensive and detailed. |
|  |  | Non-verbal Encouragement | 90 | D106 A word or an action you say or do may give others confidence, and they may trust you. D101 If you only communicate purely through language, some patients may feel a sense of estrangement. Then body language comes into play, such as eye contact during communication. In some cases, I will tell you that I am looking at you, and they will feel that our communication is barrier-free.  D22 I think, for example, the encouragement of eye contact, including possible body movements, under the condition that the patient's condition allows, at least they are not in a state of impulsively hurting others. I can give a slight pat on the shoulder, the support of eye contact, if it is beneficial to their condition, I would be willing to do. |
|  |  | Conversation Techniques | 94 | D110 It is very important to grasp the right measure in this communication skill, we call it making the complex simple. If the patient's condition is severe, we don't need to make the condition too serious, to avoid causing them excessive panic; if the condition is mild, we don't need to make it too trivial, we need to make them take it seriously but not blindly nervous, that is to say, we need to make them take it seriously, and also make them take it lightly, despise it. D106 If the patient has the desire to express but has some deviation in their thinking, we don't need to follow their wrong thinking, but with appropriate techniques, patiently guide, and slowly help them clarify their thinking. This respects the patient's expression and also helps them to see the situation more clearly. |
|  |  | Acceptable Language | 41 | D59 After clarifying the patient's condition, I explain it to them in a way they can understand, so they have a better awareness of their illness and can take better care of themselves. There's also a comforting aspect to it, as knowing their condition can give them more peace of mind. For patients with malignant tumors or those with poor prognoses, we often choose to inform their family members first. |
|  | Listening | Verbal Information | 91 | AH9 When communicating with patients, pay close attention to their expressions, carefully capturing unspoken needs and clues related to diagnosis and treatment from their language, to more accurately understand the patient. |
|  |  | Non-verbal Information | 79 | D101 Some patients with higher education levels can write, while those with lower education levels, we ask them questions, and their nods or shakes of the head are important signals. When we communicate with them, we will say, 'If you understand, nod your head or shake hands with me,' to interact in this way.  D26 If patients have corresponding body language, we will pay attention to capture it; these unspoken signals can supplement verbal expression, allowing us to understand patients more comprehensively.  D49 When you first inquire about a patient, observe their demeanor, their behavior and gestures during communication, and their facial expressions.  D65 I will pay close attention to their body language, facial expressions, and the tone or manner of speaking, to sense their current emotional state. |
|  |  | Metaphor | 66 | D111 You have to think about what the patient is not directly saying, not just listen to the surface-level expression, but also consider the possible underlying thoughts. D17 Some female patients are more reserved when speaking, and they won't be honest if asked in front of many people, they will only deny. In such cases, ask them roughly in front of many people first, and then find an opportunity to communicate with them alone, and they will be willing to share what's on their mind, and we can understand their true situation.  D11 Just like we often encounter female patients on night shifts who say they have stomach pain, but hesitate when their parents are around. Only by taking them to a separate room for a detailed inquiry do we find out it might be ectopic pregnancy, a situation that's hard to talk about openly; and some elderly patients who say they feel unwell and want to be hospitalized, but it's not really discomfort, it's because of family disputes over property, they can't say it directly, they can only dodge by saying 'unwell'. Only by understanding these unspoken meanings can we truly help the patients, and achieve what narrative medicine advocates. |
|  | Reflection | Deconstruction | 88 | D105 Everyone's experiences and situations are different, and we cannot judge solely from the perspective of a doctor, but rather slowly understand their current situation from their story. This way, we can understand that their inner hopes are reasonable and meaningful, and we can truly meet their needs and demands, which is very meaningful, rather than judging their requests from the perspective of a doctor. |
|  |  | Understanding and Analysis | 44 | D94 It's advisable to share similar experiences with patients, either from your own life or those of people you know, and communicate with them using personal feelings. This can gradually earn the patient's trust and encourage them to open up more. We can also more truly understand their current situation and inner struggles, and use this understanding to advance practice. |
|  |  | Explanation Skills | 59 | D66 When communicating with patients, you can use small analogical techniques. For example, tell them that others have encountered similar situations and have gradually improved, using these examples to draw parallels and comparisons. This can guide them to look at problems from a different angle, not to be stuck in their current emotions, and their inner confusion and unease can gradually be alleviated, which is also quite helpful for advancing practice. |
|  |  | Creative Skills | 67 | AH4 I would think, for example, why is he so anxious? Why doesn't he have family to take care of him? What has happened? Why did he get this disease? If what I'm doing is considered association, I think it just helps me better understand the patient I'm currently facing, why he is this way. It's not about judging him, it's just about knowing why this person is like this, and then I can figure out what to do. |
|  | Responding | Naming Emotions | 56 | D9 When communicating with patients, you will have a clear judgment about their state of mind. Based on the stories they tell and the feelings they express, you can gradually detect whether they are uneasy, lack motivation, or have no hope for the situation. However, I wouldn't say these things directly to the patient. Instead, I would adjust my communication style based on their state, sometimes even having in-depth conversations with their children, so that the family can also understand their current mindset. During communication, provide more positive guidance to help them gradually alleviate their discomfort. |
|  |  | Feedback | 141 | D6 In the past, when patients cried or were emotionally upset, I could comfort them with physical contact like patting shoulders or shaking hands, but after three years of the pandemic, along with the actual situation of doctor-patient relationships, I am now more cautious. However, from the patient's perspective, this kind of comforting response can actually make them feel cared for, so even though there are now some reservations, I still think about responding to their emotions in an appropriate way, not letting them feel alone. |
|  |  | Communication | 122 | D121 should first stand in his position, recognize his viewpoints and feelings in the heart. Many doctors may find the patient's demands too subjective, but in fact this subjectivity is particularly important. Moreover, using inclusive language to affirm his feelings, empathize with his emotions, can make the patient more willing to speak out what's on his mind. Secondly, it's not only necessary to objectively understand the medical issues he needs us to solve, but also to figure out his current mindset and thoughts, and to achieve emotional resonance with the patient. In this way, the patient can feel understood, and useful medical information can be captured from his expressions, making practice warmer and more directed. |
|  |  | Meeting Needs | 97 | D53 first needs to distinguish whether the patient's needs are appropriate. For example, some patients hope that the incision will be beautiful when having surgery, or breast cancer patients want to retain their breasts as much as possible. We believe these are reasonable thoughts. In such cases, we will clearly let the patient know that we are willing to do our best to help them realize these wishes, which can make the patient feel more at ease. |
|  |  | Sharing Experiences | 5 | D11 needs to find appropriate topics based on the patient's age to close the distance. If the patient is older, I will chat with him about my parents, uncles, and aunts, telling them that they have also encountered similar situations before, so that he feels more at ease; if the patient is about the same age as me, I will talk about similar situations I have encountered, or other patients' experiences. Using such examples that are close to his life for communication can make the patient feel more intimate. |

Note: D = Doctor; N = Nurse; AH = Allied Health Professional; A = Administrator. Codes refer to specific interviewees.
