# Supplementary material for: Determinants of Chinese physicians’ engagement in narrative medicine: a comprehensive SEM-ANN analysis
Source: Front Med (Lausanne). 2026 Jan 15;12:1694846. doi: 10.3389/fmed.2025.1694846 (PMC12851974; doi:10.3389/fmed.2025.1694846)
Supplement: Supplementary file 2 [file Table_2.docx]

**Supplementary Table 1**

| **Construct** | **Dimension** | **Measurement items** | **Reference** |
| --- | --- | --- | --- |
| ATT | | A1 I consider narrative medicine to be valuable. | Ajzen (1991)  Jia et al. (2021)  Guo et al. (2022)  Yang et al. (2023) |
|  |  | A2 I consider narrative medicine to be feasible. |  |
|  |  | A3 I consider the development of narrative medicine to be promising. |  |
| SN | | B1 China's healthcare policies have advocated for and supported the development of narrative medicine. | Ajzen (1991)  Cheng and Wang (2024) |
|  |  | B2 Medical institutions in China have actively promoted the practice of narrative medicine. |  |
|  |  | B3 The principles of narrative medicine have been systematically integrated into medical education in China. |  |
|  |  | B4 Patients increasingly expect healthcare services characterized by greater humanistic warmth. |  |
| PBC | | C1 I can competently perform narrative medicine work. | Ajzen (1991)  Schwarzer et al. (1999)  Cheng and Wang (2024) |
|  |  | C2 I can resolve complex challenges encountered in narrative medicine practice. |  |
|  |  | C3 I have confidence in my ability to implement narrative medicine practices. |  |
| POS | | D1 My healthcare institution implements material rewards and non-material recognition to incentivize narrative medicine practices. | Eisenberger et al. (2020)  Li et al. (2024b)  Liu et al. (2024) |
|  |  | D2 Narrative medicine practices have been incorporated into the institutional performance evaluation system. |  |
|  |  | D3 Leadership within my institution actively advocates for the implementation of narrative medicine. |  |
|  |  | D4 My institution provides dedicated supporting facilities for narrative medicine, including venue allocation, funding, and personnel resources. |  |
|  |  | D5 A culture of humanistic medicine emphasizing narrative competence has been fostered within my institution. |  |
| PIOS | | E1 I observe and emulate senior colleagues' approaches to narrative medicine in clinical settings. | Bandura and Walters (1977)  Wenger et al. (2002) |
|  |  | E2 I participate in spontaneously organized cross-institutional narrative medicine symposiums (e.g., workshops, communities of practice). |  |
|  |  | E3 Colleagues proactively share narrative medicine experiences in informal contexts (e.g., lunch breaks, WeChat groups). |  |
|  |  | E4 Clinical preceptors have demonstrated narrative medicine communication techniques during bedside teaching. |  |
|  |  | E5 I gain insights into narrative medicine through healthcare influencers' social media posts or medical blog discussions. |  |
| PB | Guidance | F1 When guiding the patient's narrative, I am good at asking open-ended questions to guide the patient to provide detailed information. | Guo et al. (2023)  Huang (2016)  Ma (2019)  Liang and Wang (2020) |
|  |  | F2 When guiding the patient, I am good at using body language to encourage narration. |  |
|  |  | F3 When guiding the patient, I am good at using conversational techniques such as repetition and summarization to guide the patient's narrative. |  |
|  |  | F4 When guiding the patient, I am good at using appropriate silences or pauses to encourage narration. |  |
|  |  | F5 When guiding the patient, I am good at using easy-to-understand language to guide the patient's story. |  |
|  | Listening | F6 When listening to patients, I am good at maintaining an unbiased and inclusive listening attitude. |  |
|  |  | F7 When listening to patients, I am good at obtaining information from the patient through language. |  |
|  |  | F8 When listening to patients, I am good at capturing information through body language, facial expressions, and tone of voice when listening to patients. |  |
|  |  | F9 When listening to patients, I am good at capturing the hidden content behind the information they tell me. |  |
|  | Reflection | F10 When reflecting on patient narratives, I am adept at analyzing the patient's main concerns from the patient's narrative. |  |
|  |  | F11 When reflecting on patient narratives, I am adept at perceiving and analyzing patients' feelings and experiences. |  |
|  |  | F12 When reflecting on patient narratives, I am good at understanding and interpreting patients' stories of illness, negative emotions. |  |
|  |  | F13 When reflecting on patient narratives, I am good at using my imagination to analyze a variety of information. |  |
|  | Response | F14 In responding to the patient's narrative, I was able to give feedback to the patient about the emotions I observed. |  |
|  |  | F15 I am able to convey respect to patients when responding to their narratives. |  |
|  |  | F16 I am able to convey understanding to patients when responding to their narratives. |  |
|  |  | F17 I am able to show support for the patient when responding to their narrative. |  |
|  |  | F18 In responding to the patient's narrative, I was able to indicate to the patient that I had understood his needs. |  |

**Abbreviations:** ATT: attitude; PBC: perceived behavioral control; SN: subjective norm; POS: Perceived Organizational Support; PIOS: Perceived Informal Organizational Support; PB: practice behavior
